# Translational repression by an RNA-binding protein promotes differentiation to infective forms in *Trypanosoma cruzi*

**DOI:** 10.1371/journal.ppat.1007059

**Published:** 2018-06-04

**Authors:** Maria Albertina Romaniuk, Alberto Carlos Frasch, Alejandro Cassola

**Affiliations:** Instituto de Investigaciones Biotecnológicas, UNSAM-CONICET, San Martín, Provincia de Buenos Aires, Argentina; Instituto Carlos Chagas, FIOCRIZ, BRAZIL

## Abstract

Trypanosomes, protozoan parasites of medical importance, essentially rely on post-transcriptional mechanisms to regulate gene expression in insect vectors and vertebrate hosts. RNA binding proteins (RBPs) that associate to the 3’-UTR of mature mRNAs are thought to orchestrate master developmental programs for these processes to happen. Yet, the molecular mechanisms by which differentiation occurs remain largely unexplored in these human pathogens. Here, we show that ectopic inducible expression of the RBP TcUBP1 promotes the beginning of the differentiation process from non-infective epimastigotes to infective metacyclic trypomastigotes in *Trypanosoma cruzi*. In early-log epimastigotes TcUBP1 promoted a drop-like phenotype, which is characterized by the presence of metacyclogenesis hallmarks, namely repositioning of the kinetoplast, the expression of an infective-stage virulence factor such as *trans*-sialidase, increased resistance to lysis by human complement and growth arrest. Furthermore, TcUBP1-ectopic expression in non-infective late-log epimastigotes promoted full development into metacyclic trypomastigotes. TcUBP1-derived metacyclic trypomastigotes were infective in cultured cells, and developed normally into amastigotes in the cytoplasm. By artificial *in vivo* tethering of TcUBP1 to the 3’ untranslated region of a reporter mRNA we were able to determine that translation of the reporter was reduced by 8-fold, while its mRNA abundance was not significantly compromised. Inducible ectopic expression of TcUBP1 confirmed its role as a translational repressor, revealing significant reduction in the translation rate of multiple proteins, a reduction of polysomes, and promoting the formation of mRNA granules. Expression of TcUBP1 truncated forms revealed the requirement of both N and C-terminal glutamine-rich low complexity sequences for the development of the drop-like phenotype in early-log epimastigotes. We propose that a rise in TcUBP1 levels, in synchrony with nutritional deficiency, can promote the differentiation of *T*. *cruzi* epimastigotes into infective metacyclic trypomastigotes.

## Author summary

*Trypanosoma cruzi* epimastigotes proliferate in the midgut of the hematophagous insect vector. Insect vectors can spend long periods of time without feeding, during which epimastigotes differentiate to infective metacyclic trypomastigotes in a process termed metacyclogenesis. This metamorphosis involves multiple phenotypic changes, involving the repositioning of the mitochondrial DNA (kinetoplast) and expression of virulence factors. Here, we show that the RBP TcUBP1 is transiently enriched during metacyclogenesis, and that ectopic expression of TcUBP1 promotes these phenotypic changes in epimastigotes, finally leading to infective metacyclic forms. Using four different approaches we found that TcUBP1 promotes translational repression together with growth arrest, both of which are characteristics of metacyclogenesis. Mechanistically, we show that low complexity regions in TcUBP1 could be involved in translational repression leading to phenotypic changes, suggesting their involvement in the formation of silenced ribonucleoprotein complexes. We conclude that TcUBP1 can act in a post-transcriptional regulatory cascade by repressing translation of multiple mRNA targets, thus promoting irreversible phenotypic changes leading to metacyclic infective forms.

## Introduction

Gene expression regulation is required to balance the synthesis of the necessary protein components that a cell needs to survive, divide or differentiate. In eukaryotes, the first layer of regulation is at the level of transcription. Beyond this layer of regulation reside several mechanisms regulating gene expression at the post-transcriptional level. The complexity of these post-transcriptional mechanisms, operating over protein-coding transcripts, covers from mRNA processing in the nucleus, to silencing in cytoplasmic foci [1]. RNA-binding Proteins (RBPs) are crucial for these processes to be achieved in a controlled fashion, recognizing specific sequences or structural motifs mainly in the non-coding 3´untranslated regions (3´-UTR) of mRNAs. There are many identifiable RNA-binding domains (RBDs), of which the RNA-Recognition Motif (RRM) is the best-characterized [2]. However, the exact function of an RBP cannot be inferred by the presence of one or more RBDs. Recent findings suggest that intrinsically disordered sequences and low complexity (LC) domains accompanying RBDs in RBPs could play an important role in protein-protein interactions, as well as in the recruitment of other proteins for the formation of ribonucleoprotein (RNP) complexes [3].

Trypanosomes have proven to be very interesting models for the study of RBPs and post-transcriptional regulation of gene expression. These are single-celled parasites affecting humans and domestic animals, in which post-transcriptional mechanisms have an enormous influence on the final outcome of gene expression [4, 5]. The genome organization of trypanosomes and related kinetoplastid organisms is highly unusual: intron-less protein-coding genes are organized into large polycistrons. A few transcription start sites with no canonical RNA polymerase II promoter sequences are in charge of transcription initiation. The synthesis of the polycistron is concomitant with its processing by *trans*-splicing and polyadenylation, giving rise to mature monocistrons. Thus, multiple open reading frames, unrelated in function, are synthesized. As such, post-transcriptional mechanisms such as degradation, silencing and translation efficiency of mRNA seem to be the central events in the regulation of gene expression in trypanosomes [5, 6]. *Trypanosoma cruzi* is the parasite responsible for Chagas disease in the Americas. This protist presents a complex life cycle, alternating between a vertebrate host and an insect vector [7]. These dissimilar environments force *T*. *cruzi* to accomplish differentiation processes to cope with different nutrients, immune responses and temperatures. Metacyclogenesis is the process by which non-infective replicating epimastigotes develop into infective non-replicating metacyclic trypomastigotes in the hindgut of the insect. This process is characterized by the repositioning of the mitochondrial DNA (Kinetoplast) towards the posterior region of the cell body [8]. After a blood meal, metacyclic trypomastigotes are released together with the faeces, allowing them to infect the host, either through mucous membranes or through small abrasions produced from scratching. After cellular infection of the host, metacyclic trypomastigotes are released from the parasitophorous vacuole, allowing them to differentiate into amastigotes, which replicate in the cytoplasm. After a defined number of divisions, amastigotes differentiate into trypomastigotes, which are released from the infected cell, allowing them to infect new cells. Alternatively, trypomastigotes can be ingested by an insect vector in a blood meal, which then develop into non-infective replicating epimastigotes in the insect, closing the cycle [7].

How trypanosomes accomplish these transformation processes without transcription initiation regulation at every single gene has been a matter of study by many research groups. Several lines of evidence point towards the role of RBPs in regulating the beginning of developmental programs to which trypanosomes commit at the differentiation outset [9–12]. In general, most experimental approaches have been able to demonstrate the impact of RBPs on mRNA abundance, suggesting roles as modulators of mRNA stability [13, 14]. However, only recently we are beginning to understand the effect of RBPs on the translational modulation of mRNA in trypanosomes [15, 16]. In this line of evidence, trypanosomes have shown to respond to starvation stress by shutting down protein synthesis and protecting mRNAs from degradation through their recruitment to mRNA granules [17, 18]. TcUBP1 is one of the best characterized proteins from starvation-induced mRNA granules in *T*. *cruzi*, and is one of the most characterized RBPs in trypanosomes [17, 19–21]. In this work, we show the growth phase-dependent phenotypic result of the ectopic inducible expression of this single RRM-containing RBP. TcUBP1 expression in early-log epimastigotes promoted a drop-like phenotype with several characteristics of epimastigotes undergoing metacyclogenesis. However, when TcUBP1 was induced in late-log epimastigotes we obtained complete progression to infective metacyclic trypomastigotes. Functional characterization of TcUBP1 by tethering it to the 3’ UTR of a reporter mRNA, unveiled its role as a translational repressor. Furthermore, we demonstrate that TcUBP1-induced differentiation process is dependent on both the N and the C-terminal Q-rich LC sequences, highlighting the influence of these uncharacterized domains.

## Results

### An increase in TcUBP1 levels promotes the development of metacyclogenesis hallmarks

**Fig 1:**
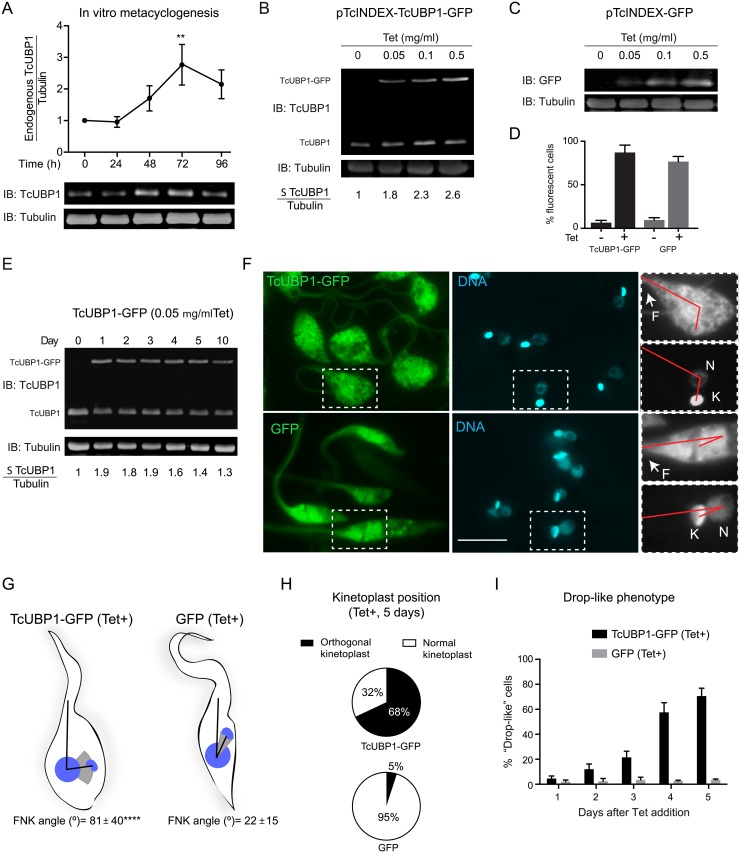
TcUBP1-GFP ectopic expression in epimastigotes. (A) Wt epimastigotes were incubated in TAU-3AAG medium and TcUBP1 expression was assessed by Western blot for four days. Tubulin blot served as loading control. Values represent mean ± SD of 3 independent replicates. TcUBP1/Tubulin before TAU incubation was considered as 1. A representative Western blot experiment is shown. **P< 0.01 by Anova-Dunnett test. (B) Levels of TcUBP1-GFP induced expression 48 hours after the addition of different concentrations of Tet determined by Immunoblot (IB), and compared to TcUBP1 endogenous levels using an anti-TcUBP1 antibody. The ratio between the sum (Σ) of TcUBP1 forms (endogenous and ectopic) and Tubulin before Tet addition was considered as 1. (C) Levels of GFP induced expression at different Tet concentrations detected with an anti-GFP antibody and compared to Tubulin levels. (D) Percentage of GFP positive epimastigotes before and after induction with Tet determined by flow cytometry. Values represent mean ± SD of 6 independent replicates. (E) Immunoblot from a time course analysis of TcUBP1-GFP induced expression using 0.05 μg/ml Tet, together with endogenous TcUBP1. Tubulin was used as a loading control. The ratio between the sum of TcUBP1 forms (endogenous and ectopic) and Tubulin before Tet addition was considered as 1. (F) Fluorescence microscopy of epimastigotes expressing TcUBP1-GFP, or GFP, after 5 days of induction. Magnifications highlight a red line connecting the base of the flagellum, the nucleus and the kinetoplast, evidencing the repositioning of the kinetoplast. DNA was stained with DAPI, shown in cyan. Images are representative of 6 independent experiments. Scale bars, 5 μm. (G) Parasites schemes represent approximate forms obtained from induced TcUBP1-GFP or GFP cultures. In these parasites, the angle formed by the flagellum, the nucleus and the kinetoplast (FNK angle) was measured. The mean FNK angle is shown with black lines, and the kinetoplast is placed in this mean position. The gray spectrum represents SD above and below the mean FNK angle. ****P<0.0001 by T test (TcUBP1 N = 64, GFP N = 58). (H) Pie chart representing the mean percentages of cells with orthogonal or normal kinetoplast position in parasites induced for the expression of TcUBP1-GFP or GFP after 5 days. Values were obtained from 4 independent experiments. (I) Time course quantitation of parasites displaying drop-like phenotype in induced TcUBP1-GFP and GFP cultures.

The transformation of non-infective epimastigotes into infective metacyclic trypomastigotes (metacyclogenesis) involves changes in the pattern of expressed genes, resulting in important morphological and functional differences between these developmental forms of *T*. *cruzi* [22]. This differentiation process can be stimulated *in vitro* by incubating epimastigotes from a late logarithmic phase of growth in a chemically defined medium with the given name of Triatomine Artificial Urine (TAU), followed by an incubation in TAU supplemented with glucose and amino acids [8]. Fully developed metacyclic trypomastigotes emerge after 96 hs of incubation. While assessing TcUBP1 levels during TAU *in vitro* metacyclogenesis of wild type (wt) non-infective replicative epimastigotes to infective non-replicative metacyclic trypomastigotes, we found that TcUBP1 protein levels increased significantly after 72 h of differentiation onset (Fig 1A). To reduce the burden of complexity of the differentiation process, and to better understand the effect of the rise in TcUBP1 levels, we made use of a Tetracycline (Tet)-based inducible system [23] to express TcUBP1-GFP ectopically in non-infective epimastigotes. We decided to use a GFP-fusion protein to allow easy tracking of recombinant protein expression, considering that TcUBP1-GFP fusion has the same dynamic localization patterns as endogenous TcUBP1 [17, 19]. GFP by itself was expressed in the same way to serve as a negative control. A concentration of 0.05 μg/ml Tet was sufficient to generate a detectable increase in the levels of TcUBP1-GFP and GFP (Fig 1B and 1C). At this level of expression, TcUBP1-GFP was distributed in the cytoplasm and nucleus (S1 Fig, top panel), as expected [17, 19]. However, higher Tet concentrations promoted preferential TcUBP1-GFP accumulations in the nucleus (S1 Fig, bottom panel). In these parasites, cytoplasmic TcUBP1-GFP was lower than in parasites induced with a lower concentration of Tet. We have previously documented the nucleocytoplasmic nature of TcUBP1 by nuclear accumulation under arsenite stress [19, 24]. We suggest that excessive protein concentration in the nucleus is likely to be the result of TcUBP1 that is not engaged in RNP complexes. This observation argues for a stringent regulation of TcUBP1 levels in the cytoplasm. Because of this, we continued our experiments with parasites expressing TcUBP1-GFP at low Tet concentrations (0.05 μg/ml), which would reflect a protein localization that is similar to the wt protein. Flow cytometric analysis showed low expression leakage in uninduced cells, and a high percentage of fluorescent cells after Tet addition (Fig 1D). Expression of TcUBP1-GFP could be detected 24 hs after induction. At this level of expression, the level of total TcUBP1 (endogenous plus ectopic) was 80–90% higher than endogenous TcUBP1 level in uninduced parasites (Fig 1E). TcUBP1-GFP levels remained constant for 5 days, after which Tet was re-added (Fig 1E). Strikingly, induced ectopic expression of TcUBP1-GFP in parasites from an early logarithmic phase of growth promoted a shift in the position of the kinetoplast as compared to induced GFP parasites (Fig 1F, compare upper and bottom panels). In TcUBP1-GFP parasites we observed an orthogonal kinetoplast related to the axis that is formed by the flagellum and nucleus (Fig 1F, upper panel). In order to quantitate this effect, we measured the angle that is formed between the flagellum and the kinetoplast using the center of the nucleus as the vertex, hereafter FNK angle. In parasites ectopically expressing TcUBP1-GFP we obtained a mean FNK angle of 81° with a standard deviation (SD) of 40°, while parasites expressing GFP displayed a mean FNK angle of 21° with a SD of 15° (Fig 1G, S2 and S3 Figs, S1 Table), showing a statistically significant difference (p<0,0001). This kinetoplast disposition is similar to that observed in intermediate forms of wt parasites under the first 24–48 h of *in vitro* metacyclogenesis [22, 25] (S4A Fig), or in the parasites developing in the rectum of the insect vector *Triatoma infestans* [25]. In some parasites, repositioning of the kinetoplast almost surpassed the nucleus, showing a similar position of the kinetoplast in parasites at 48 h of *in vitro*-induced metacyclogenesis (magnification Fig 1F, S4A and S4B Fig). This particular phenotype was noticed in 68% of TcUBP1-GFP expressing parasites, being practically absent in GFP expressing cells (5%) (Fig 1H, S2 and S3 Figs).

TcUBP1-GFP expressing parasites also showed a shape change, with a wider and rounded cell body as compared to parasites expressing GFP (Fig 1F). We used the term drop-like phenotype for these parasites, as previously defined by Kollien and Schaub for epimastigotes with this morphology [26]. Drop-like forms have been considered as naturally-occurring insect-dwelling intermediate forms between epimastigotes and metacyclic trypomastigotes, having a round posterior end and a kinetoplast beside or at the posterior side of the nucleus [25]. Drop-like parasites expressing TcUBP1-GFP could easily be noticed two days after Tet addition, comprising the majority of the population from day four onwards (Fig 1I). In our hands, parasites with a drop-like phenotype could be detected from the first 24h of *in vitro* metacyclogenesis (S4C Fig), comprising the majority of the population at the end of the incubation, thus suggesting that these forms can be observed during the differentiation process to infective forms. We would like to highlight that the development of these phenotypic changes only takes place when TcUBP1-GFP is located in the cytoplasm, and not when TcUBP1-GFP accumulates in the nucleus at higher Tet levels (S1 Fig). This observation argues for a cytoplasmic role of TcUBP1 in promoting this phenotype. In order to discard an unspecific differentiation stimulus due to ectopic expression, we analyzed if the expression of another RRM-type RBP could also induce these phenotypic changes. For this, we ectopically induced the expression of TcRBP35-GFP (TcCLB.510661.230) [27], which was found to be transiently enriched during metacyclogenesis by a differential proteomic approach [28]. No phenotypic changes were found after 5 days of expression of this RBP (S5 Fig), suggesting that induced ectopic expression of any RBP does not induce spontaneous unspecific differentiation. Overall, these results strongly suggest that ectopic expression of TcUBP1 in early-log epimastigotes leads to a phenotypic alteration with the hallmarks of *T*. *cruzi* metacyclogenesis.

**Fig 2:**
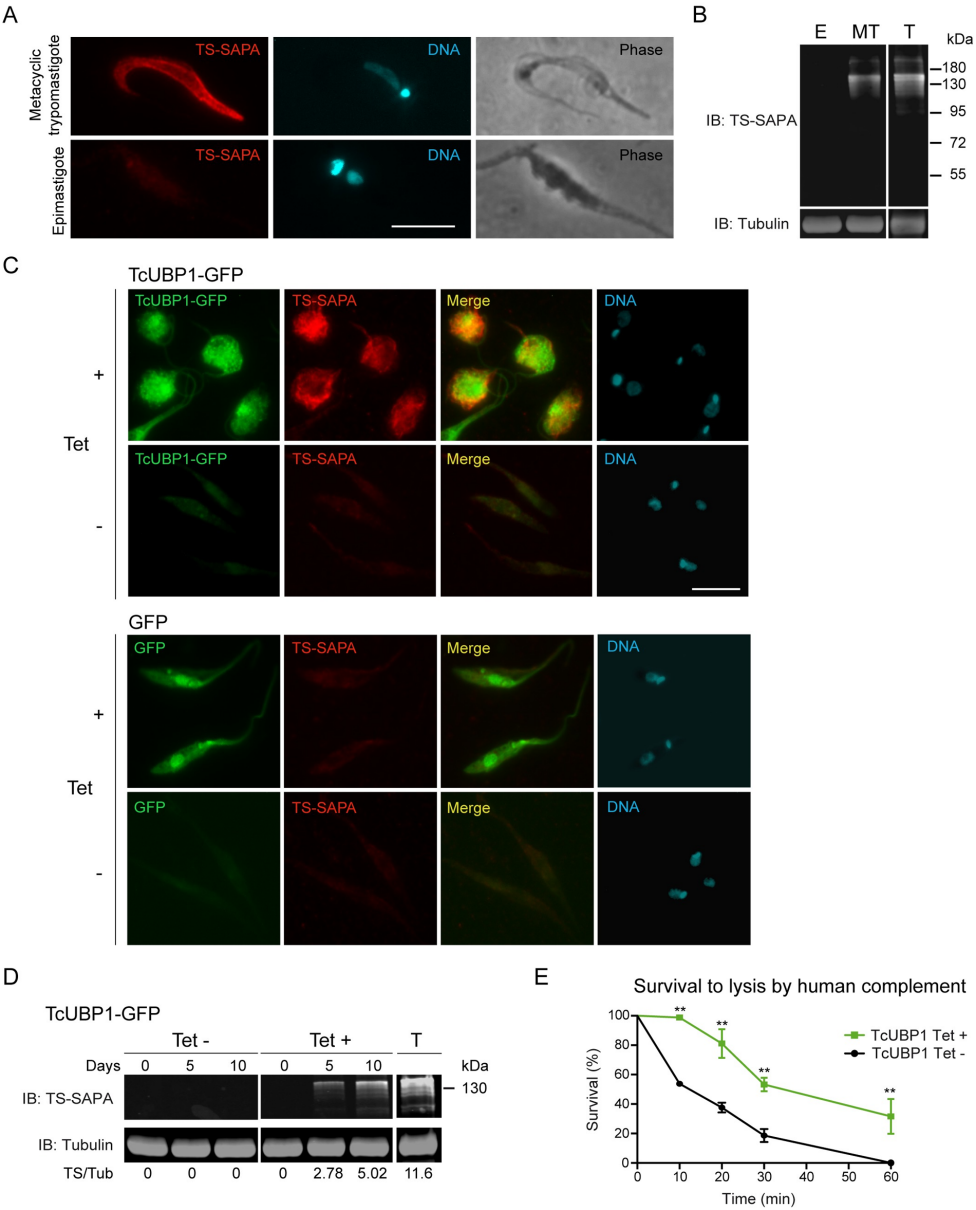
Induced ectopic expression of TcUBP1-GFP in early-log phase epimastigotes promotes the development of metacyclic trypomastigotes features. (A) Immunofluorescence of infective metacyclic trypomastigotes showing staining with anti-TS-SAPA antibody, and non-infective epimastigotes showing no staining. (B) Western blot of epimastigotes (E) and metacyclic trypomastigotes (MT) protein extracts with the anti-TS-SAPA antibody. An extract from cell-derived trypomastigotes (T) served as a positive control. Tubulin immunoblot was used as loading control. (C) Staining of TS-SAPA by immunofluorescence in induced (10 days) or uninduced early-log epimastigotes expressing TcUBP1-GFP or GFP. DNA was stained with DAPI. Images are representative of 3 independent experiments. (D) Western blot of uninduced or induced epimastigote protein extracts ectopically expressing TcUBP1-GFP. As a positive control for the anti-TS-SAPA antibody we used a protein extract from cell-derived trypomastigotes (T). Tubulin blot served as a loading control. Image is representative of 4 independent experiments. Scale bars, 5 μm. (E) Survival of epimastigotes to lysis by human complement. TcUBP1-GFP uninduced or induced parasites were incubated in PBS supplemented with 10% fresh human serum for the defined time course. Microscopic analysis was performed at each time point without fixation, only motile parasites were considered alive. **P< 0.01 by Two-way Anova- test.

This putative differentiation event in parasites ectopically expressing TcUBP1-GFP made us wonder if it was concomitant with the expression of infective-stages biomarkers. To answer this, we assessed the expression of one of the most characterized virulence factors from *T*. *cruzi*, *trans*-sialidase containing the Shed Acute Phase Antigen repeats (TS-SAPA) [29]. TS-SAPA is a marker expressed by metacyclic trypomastigotes and blood trypomastigotes [30], and not by epimastigotes [31] (Fig 2A and 2B). TcUBP1-GFP expressing parasites, displaying orthogonal kinetoplast and drop-like phenotype, were positive for TS-SAPA staining (Fig 2C), while control parasites were not stained (Fig 2C). Maximal TS-SAPA staining was observed after 10 days of induction (Fig 2C). Induced and uninduced control parasites expressing GFP showed no staining (Fig 2C). Given the high proportion of parasites expressing TcUBP1-GFP, we could also detect the expression of TS-SAPA by Western blot (Fig 2D), showing a similar band pattern as cell-derived trypomastigotes (T), used as a positive control in this experiment. The obtained band pattern is the consequence of the expression of multiple genes [32]. It is noteworthy that uninduced TcUBP1-GFP transgenic parasites showed no expression of TS-SAPA, either by immunofluorescence or Western blot, reflecting that expression is promoted by the rise of TcUBP1-GFP levels. To further push the characterization of drop-like forms, we tested their resistance to lysis by human complement. This feature is only found in trypomastigotes (metacyclic or cell-derived) and amastigotes, and not in epimastigotes, through the expression of a variety of complement regulatory proteins [33]. We found a statistically significant increased resistance to lysis by complement in TcUBP1-GFP expressing drop-like parasites as compared to uninduced parasites (Fig 2E). This result suggests that TcUBP1-GFP expression also promotes the readiness to encounter an innate immune response from a host. Trypomastigote Small Surface Antigen (TSSA), a molecule only expressed by cell-derived trypomastigotes [34] (S6A Fig), was not detected in TcUBP1-GFP or GFP-induced parasites (S6B Fig), as expected. The differences between the expressed protein markers and the resistance to complement-mediated lysis suggests that drop-like forms could correspond to developmental intermediates of metacyclogenesis, and that TcUBP1-GFP expression is not triggering unregulated gene expression. Altogether, these results suggest that ectopic expression of TcUBP1 in parasites in early logarithmic phase of growth promotes a differentiation process with the hallmarks of metacyclogenesis.

### Metacyclic trypomastigotes obtained by TcUBP1-GFP ectopic expression are infective

**Fig 3:**
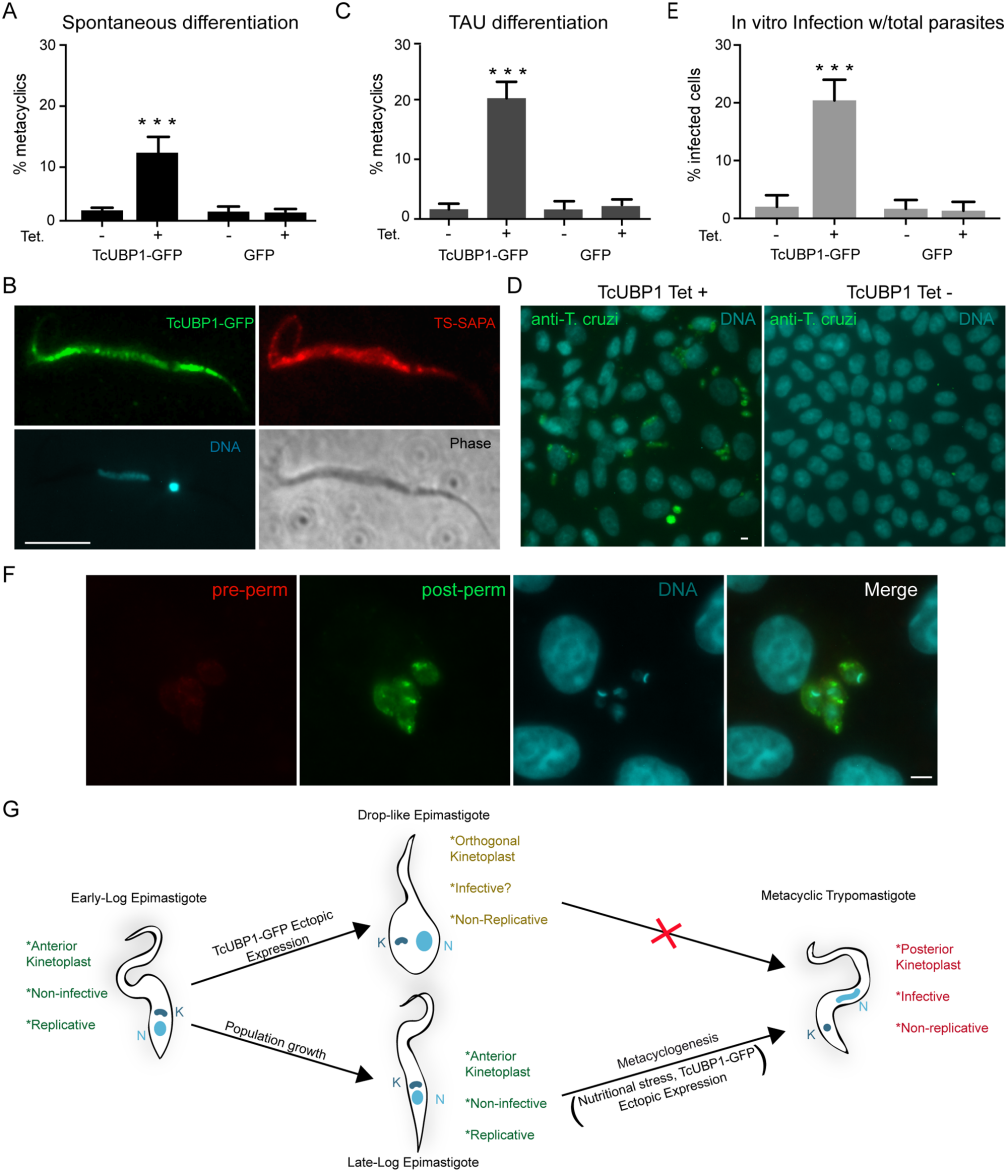
TcUBP1-GFP ectopic expression in late-log phase epimastigotes promote full metacyclogenesis. (A) The percentage of metacyclic trypomastigotes was determined in uninduced or induced TcUBP1-GFP or GFP late-log epimastigotes cultures 5 days after addition of Tet. Values represent mean ± SD of 4 independent replicates. (B) TS-SAPA staining was assessed in metacyclic trypomastigotes derived from TcUBP1-GFP induced cultures (5 days). Images are representative of 3 independent experiments. (C) The percentage of metacyclics was determined in uninduced or induced TcUBP1-GFP or GFP late-log epimastigotes incubated in TAU-3AAG medium for 96 hs. (D) Staining for intracellular amastigotes in VERO cells 7 days after incubating with induced and uninduced TcUBP1-GFP cultures. (E) Percentage of infected VERO cells 7 days post-infection with parasites derived from induced or uninduced TcUBP1-GFP or GFP cultures. Values represent mean ± SD of 3 independent replicates. (F) Infected VERO cells with intracellular amastigotes 7 days post infection with TcUBP1-GFP cultures. Amastigotes were stained with mouse anti-*T*. *cruzi* before permeabilization (red) and with rabbit anti-*T*. *cruzi* after permeabilization (green). DNA was stained with DAPI, shown in cyan. Images are representative of 3 independent experiments. ***P< 0.001 by Anova-Tukey test. Scale bars, 5 μm. (G) Model for TcUBP1-induced morphological differentiation.

TcUBP1-GFP ectopic expression in early logarithmically growing parasites promotes many of the characteristics of the developmental differentiation process from non-infective epimastigotes to infective metacyclic trypomastigotes (Figs 1 and 2). However, these parasites did not progress completely to the metacyclic trypomastigote form. Given that metacyclogenesis in *T*. *cruzi* requires a nutritional stress response [35], we induced the expression of TcUBP1-GFP in epimastigotes at late logarithmic phase of growth. This condition, which is more favorable for differentiation due to nutritional stress, resulted in the spontaneous development of metacyclic trypomastigotes at a level of 10-fold when compared to control cultures after 5 days of induction (Fig 3A). Given that ectopic expression using pTcINDEX can already be detected after 24 h of induction, this period of Tet-induction is consistent with the time course of a TAU-induced metacyclogenesis (S4A Fig). Metacyclic trypomastigotes generated *in vitro* after TcUBP1-GFP induction featured a kinetoplast at the posterior end of the cell, an elongated nucleus shape (Fig 3B), metacyclic-like motility and they did not adhere to glass, all of which are characteristic features of metacyclic trypomastigotes. TcUBP1-GFP derived metacyclic trypomastigotes also expressed TS-SAPA (Fig 3B), as expected. To further push parasites expressing TcUBP1-GFP into differentiation, we performed *in vitro* metacyclogenesis in TAU-3AAG medium. A significant difference in the number of metacyclic trypomastigotes was once again observed in TcUBP1-GFP induced cultures as compared to control uninduced or GFP expressing parasites (Fig 3C). It is important to point out that the CL Brener stock of parasites used in this work is one of the strains with the lowest capacity to differentiate to metacyclic trypomastigotes [36], thus explaining the low efficiency of this method.

As an alternative way to analyze the spontaneous development of metacyclic trypomastigotes from epimastigotes, we performed infection of VERO cells using the same number of total parasites (epimastigotes plus metacyclic trypomastigotes) after induction of TcUBP1-GFP or GFP. We found 20% of infected cells using TcUBP1-GFP expressing parasites, while we only observed 2–3% of infected cells when using uninduced control parasites (Fig 3D and 3E). Uninduced and induced GFP-expressing control parasites showed reduced (2–3%) infection rates (Fig 3E, S7 Fig), basically because in these cultures there are almost no metacyclic trypomastigotes, just epimastigotes. From this result we can make two conclusions; on one hand, that the spontaneous development of metacyclic trypomastigotes induced by TcUBP1-GFP expression can be detected by a method that is independent of the subjective discrimination between epimastigotes and metacyclic trypomastigotes by their morphology; on the other hand, that TcUBP1-GFP induced metacyclic trypomastigotes are infective *in vitro*, confirming their identity as a real developmental stage form. To confirm that the observed amastigotes were inside the cells, and not attached to the cell surface, we performed a double immunofluorescence assay with differential staining before and after permeabilization, which showed that amastigotes were indeed inside the cells (Fig 3F), as expected. We can conclude that TcUBP1 ectopic expression in early-log epimastigotes promotes a drop-like phenotype, with similar characteristics to metacyclogenesis intermediate forms (Fig 3G); whereas late-log epimastigotes (high density populations undergoing nutritional stress) respond to ectopic expression of TcUBP1 by developing into infective metacyclic trypomastigotes (Fig 3G).

### TcUBP1 reduces protein translation and promotes formation of mRNA granules

**Fig 4:**
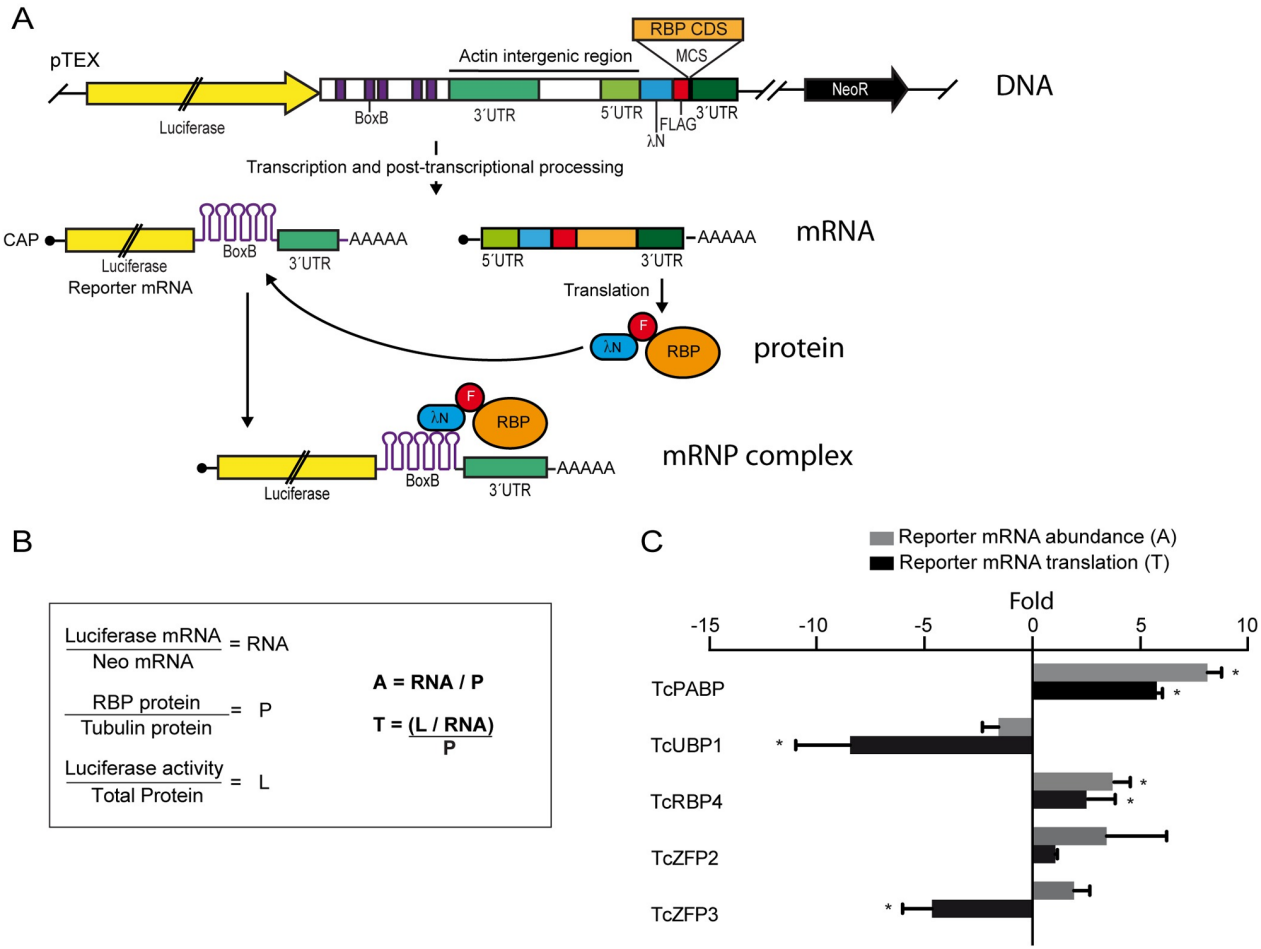
Tethering of *T*. *cruzi* RBPs to reporter mRNA. (A) Scheme of the DNA construct cloned in the pTEX vector. See Methods section for details. (B) Normalization of reporter mRNA and luciferase activity values. See Methods section for details. RNA: normalized luciferase mRNA, P: normalized RBP protein, L: normalized luciferase activity, A: reporter mRNA abundance, T: reporter mRNA translation. (C) *Trypanosoma cruzi* epimastigotes were transfected with the reporter construct in which different RBPs (TcPABP1, TcUBP1, TcRBP4, TcZFP2 and TcZFP3) were artificially tethered to the 3´ UTR of the reporter gene. The results indicate folds over values obtained with GFP as the tethered protein. Folds higher than 2 were considered significative (*). Results are shown for 3 or 4 independent experiments.

Previously, we have proposed that TcUBP1 can decrease the half-life of SMUG mRNAs *in vivo* [20], suggesting that it plays a destabilizing effect on this mRNA. The effect of TcUBP1 on epimastigote differentiation shown in this work could be mediated by a decrease in mRNA abundance due to enhanced degradation, or by an effect on mRNA translation. To answer this, we developed a versatile reporter system to tether proteins to a reporter mRNA in *T*. *cruzi* epimastigotes using the λN-BoxB approach. For this system we made use of the trypanosome’s capacity of combined polycistronic transcription and post-transcriptional processing to obtain two mature RNA molecules from a single DNA construct (Fig 4A). As a reporter mRNA we used the coding sequence for Firefly Luciferase. In the 3’ UTR of this cistron we introduced five BoxB tracts. In the second cistron we introduced the coding sequence of the 22 amino acid RNA-binding domain of λ bacteriophage antiterminator protein N (λN), followed by a Flag tag and a multiple cloning site, that would allow us to clone the sequence coding for the RBP of interest (Fig 4A). BoxB structures are recognized by λN, thus allowing the tethering of λN fusions to the 3’ UTR of the reporter mRNA [37, 38]. Furthermore, this *in vivo* system also allows determining if the tethered RBP can modulate mRNA abundance and/or translation by measuring luciferase mRNA levels and luciferase activity, respectively. The normalization applied to the obtained values is shown in Fig 4B.

For baseline measurements we used λN-GFP tethered to the 3’ UTR of luciferase reporter mRNA. This allowed us to compare the results obtained with different RBPs to those obtained with GFP. As a control of performance for our system we analyzed the effect of TcPABP1, whose ortholog has been previously characterized in a similar system in *T*. *brucei* [39]. As expected, TcPABP1 promoted mRNA stability and translation efficiency (Fig 4C), resembling the behavior observed in an analog system used in *T*. *brucei* [39]. TcUBP1 only showed a 1.9-fold decrease in reporter mRNA abundance (Fig 4C). However, we found that it could induce an 8-fold reduction in translation efficiency of the reporter mRNA, suggesting that TcUBP1 could be a potent translational repressor. To further test the system, we also included other RBPs (TcRBP4 from the RRM family group, and TcZFP2 and TcZFP3 from the CCCH Zinc Finger Family), for which a functional characterization is lacking in *T*. *cruzi*. TcRBP4 increased both mRNA abundance and translation almost four-fold when compared to GFP, suggesting a role as an mRNA stabilizer and a translational promoter (Fig 4C). TcZFP2 showed highly variable results on mRNA abundance, while it did not show an effect on translation (Fig 4C). TcZFP3 promoted an increase on mRNA abundance, which did not prove to be statistically significant (Fig 4C). However, it did promote a decrease of 4-fold in reporter mRNA translation when compared to GFP, suggesting that TcZFP3 might be a translational repressor as TcUBP1 (Fig 4C). In summary, these results are in concordance with the proposed role for TcPABP1, thus validating this *T*. *cruzi in vivo* tethering system. It allowed us to determine the putative translational repression feature of TcUBP1, making it a valuable diagnostic tool to functionally characterize RBPs in *T*. *cruzi*.

**Fig 5:**
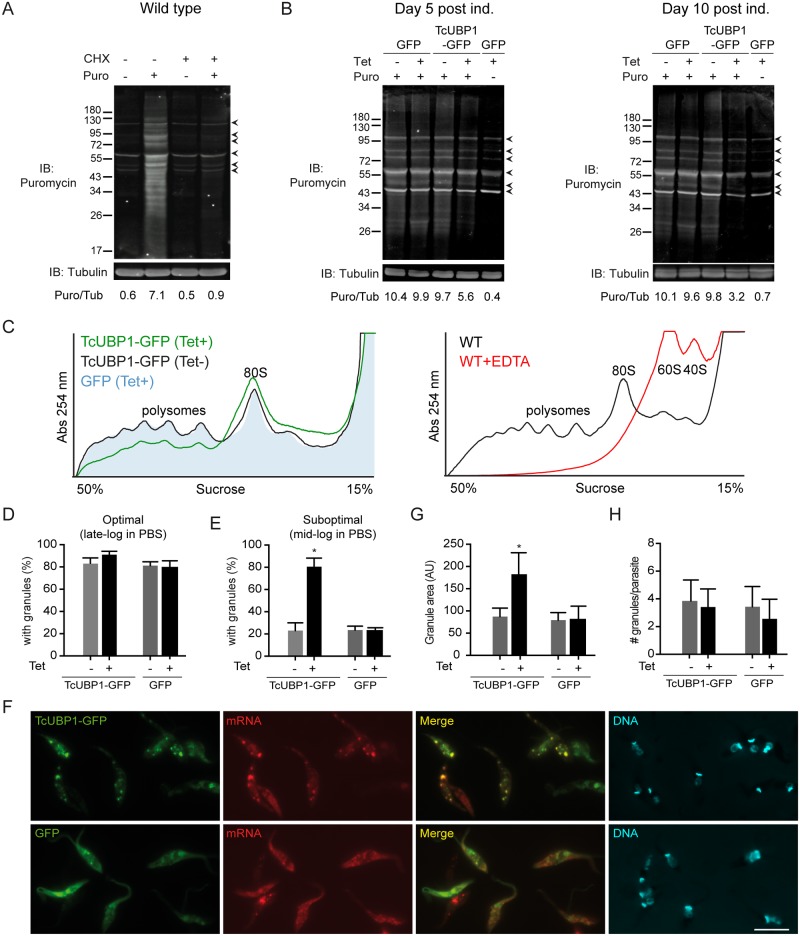
Translation repression in parasites ectopically expressing TcUBP1. (A) Wt parasites were incubated with CHX (50 μg/ml) or vehicle and then with puromycin (10 μg/ml) or vehicle for 30 minutes. Puromycin incorporation was determined by Immunoblot (IB) using an anti-puromycin antibody. Protein loading was monitored using an anti-tubulin antibody. Image is representative of 3 independent experiments. Proteins unspecifically detected by the anti-puromycin antibody in the absence of puromycin, are pointed with arrowheads. (B) GFP or TcUBP1-GFP induced or GFP uninduced parasites were cultured for 5 and 10 days. 3 x 10^7^ parasites were incubated with puromycin (10 μg/ml) or vehicle for 30 minutes. Puromycin incorporation was determined by immunoblot using an anti-puromycin antibody. Protein loading was monitored using an anti-tubulin antibody. The ratio between Puromycin fluorescence/Tubulin fluorescence (Puro/Tub) is shown in arbitrary units (AU). Images are representative of 4 independent experiments. Unspecific bands are pointed with arrowheads. (C) Left panel, polysome profiles of induced (green line) and uninduced TcUBP1-GFP (black line), and from induced GFP parasites (filled light blue profile) after 5 days of induction, in the presence of CHX. Right panel, a control polysome profile from wt parasites using CHX is shown (black line), together with the disruption of 80S ribosomes into 60S and 40S ribosomal subunits with EDTA as a specificity control (red line). (D) Percentage of parasites displaying mRNA granules under optimal starvation conditions. (E) Percentage of parasites displaying mRNA granules under suboptimal starvation conditions. (F) Microscopic images of epimastigotes expressing TcUBP1-GFP, or GFP, under suboptimal starvation conditions, together with poly(A) mRNA detection by FISH and staining of DNA with DAPI, shown in cyan. Images are representative of 5 different experiments. (G) Analysis of granule area. (H) Analysis of the number of mRNA granules per parasite. Values represent mean ± SD of 5 independent replicates. *P< 0.05 by Anova-Dunnett test. Scale bar, 5 μm.

In order to confirm the, up to now, unnoticed function of TcUBP1 as a translational repressor, we used three additional approaches. First, we made use of the SUnSET method [40], in which puromycin is used in very low amounts for a short time period, and whose incorporation in neosynthesized proteins directly reflects the rate of mRNA translation *in vivo*. To validate this system, we verified the puromycin labeling of nascent polypeptides in wt parasites, and confirmed that puromycin is not incorporated when translation is completely inhibited by cycloheximide (CHX) (Fig 5A). Early-log epimastigotes ectopically expressing TcUBP1-GFP or GFP for 5 or 10 days were incubated with puromycin, and its incorporation was detected by immunoblot with the anti-puromycin antibody. Puromycin incorporation was reduced in a time-dependent manner in parasites expressing TcUBP1-GFP (Puromycin/Tubulin ratio = 5.6), but not in uninduced or GFP-induced parasites (Puromycin/Tubulin ratio = 9.7 and 9.9, respectively) (Fig 5B). Puromycin incorporation was still observed in TcUBP1-GFP-expressing parasites five days after Tet addition, suggesting that the normal turnover of proteins required to survive was not affected in these cells (Fig 5B). As a control of complete translation inhibition we used CHX, which dropped this ratio to 0.4. Analysis of Puromycin/Tubulin ratio at 10 days after induction emphasized the effect of induced TcUBP1-GFP. Comparing CHX and induced TcUBP1-GFP induction we can conclude that TcUBP1-GFP expression did not completely block translation. We can suggest a role of TcUBP1 as a repressor of translation, operating selectively on certain mRNAs, or globally, by partially reducing translation rates of most mRNAs, but allowing the translation of mRNAs whose protein products will define the metacyclic trypomastigote or drop-like forms. These results, confirm our previous observation of the capability of TcUBP1 to repress translation in the *in vivo* tethering system, thus supporting a putative role for this RBP in the repression of protein translation in *T*. *cruzi*.

Second, we determined the translational status of the parasites by analyzing their polysome profiles. Using 15–50% sucrose gradients we separated ribosome subunits (60S and 40S) and monosomes (80S), which are not actively involved in protein synthesis, and polysomes, which are multiple ribosomes loaded onto single mRNA molecules and thus are an indication of active protein synthesis. When comparing the profile from induced TcUBP1-GFP cells to control profiles (uninduced TcUBP1-GFP and induced GFP cells) we found an overall reduction on the polysomal fraction as compared to control profiles (Fig 5C). This was concomitant with an increase in monosomes, indicating translational repression [41]. The comparison of induced GFP and uninduced TcUBP1-GFP polysome profiles showed no differences, suggesting that the induced expression from the pTcINDEX vector is not affecting the translational status of the cells (Fig 5C). A control profile using EDTA showed the disruption of 80S ribosomes into 60S and 40S ribosomal subunits, serving as a control of complete translational inhibition. These results suggest that the translational status in TcUBP1-GFP expressing parasites is reduced, although not inhibited, as compared to control parasites.

Finally, we evaluated mRNA granule formation in transfected parasites by fluorescence in situ hybridization. It is known that mRNA granules develop in response to starvation stress in *T*. *cruzi* epimastigotes [17]. Transcripts stored in granules are translationally silenced and protected from degradation, awaiting favorable nutritional conditions to resume translation [42]. In *T*. *cruzi*, an optimal starvation stimulus typically promotes the formation of mRNA granules in 80–100% of an untransfected population (Fig 5D) [17]. Because of this, we analyzed formation of mRNA granules in epimastigotes expressing TcUBP1-GFP or GFP under suboptimal starvation stimulus (see Materials and methods). In this experiment we applied the suboptimal starvation stress 36 h after addition of Tet, not allowing parasites to acquire the drop-like phenotype. The suboptimal starvation stimulus promoted the formation of mRNA granules in 20% of uninduced or GFP induced control parasites, while ectopic expression of TcUBP1-GFP promoted mRNA granules in 80% of induced parasites (Fig 5E and 5F). Also, in TcUBP1-GFP induced cultures the biggest mRNA granule from each cell was almost twice as big as those from uninduced or GFP induced control parasites (Fig 5G), while the number of granules per cell was unaltered in the four populations (Fig 5H). These results support the role of TcUBP1 as a translational repressor by enhancing the condensation and silencing of mRNA in granules under suboptimal starvation stress.

### Ectopic expression of TcUBP1-GFP promotes irreversible growth arrest

**Fig 6:**
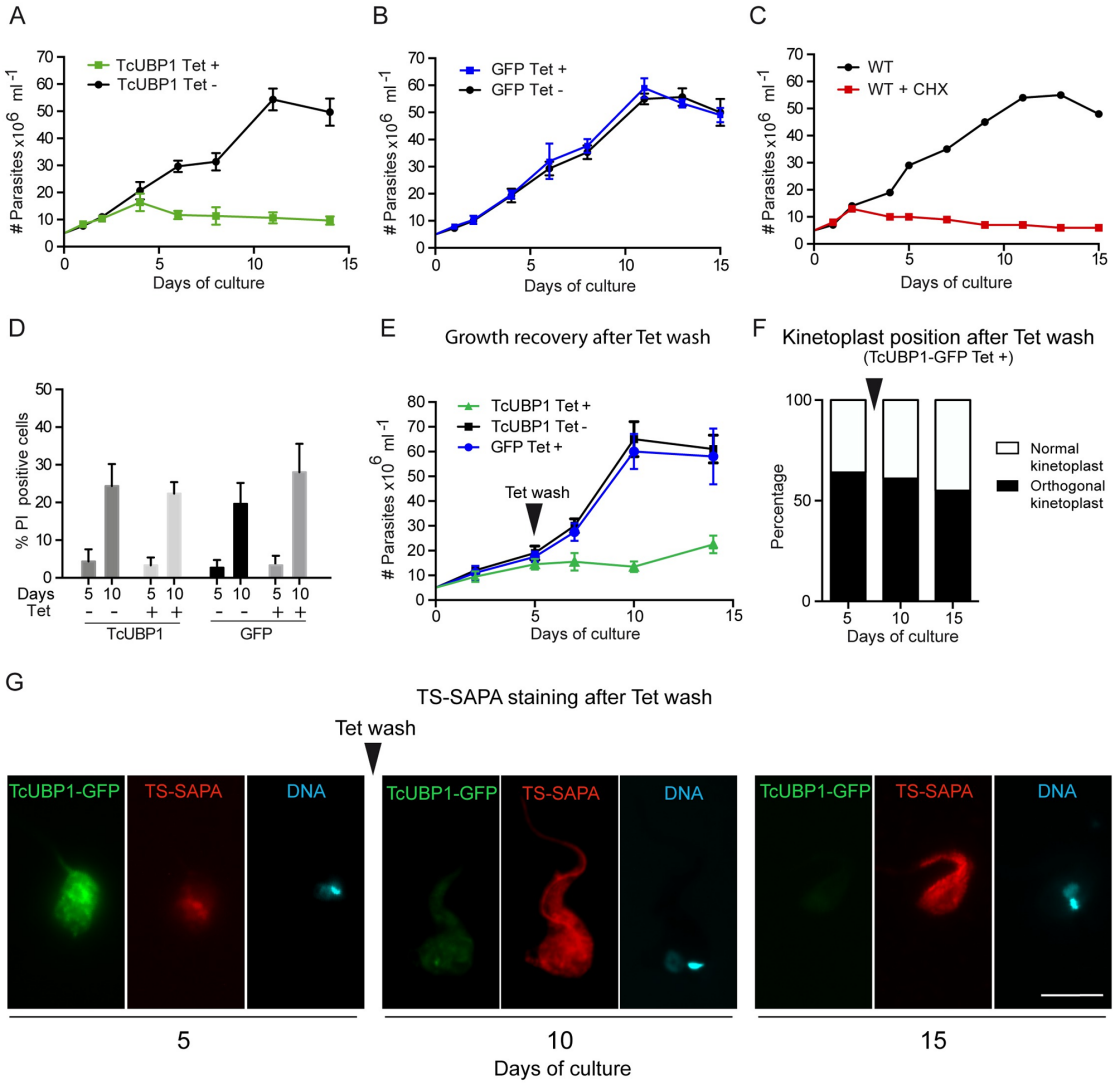
Irreversible growth arrest in parasites ectopically expressing TcUBP1. (A and B) Growth curves of induced and non-induced epimastigotes expressing TcUBP1-GFP or GFP. Values represent mean ± SD of 5 independent replicates. (C) Growth curves of wt epimastigotes in the presence or absence of CHX. Values represent mean ± SD of 3 independent replicates. (D) The percentage of dead cells was determined by flow cytometry by incubating parasites with propidium iodide at 5 and 10 days after Tet addition. Values represent mean ± SD of 4 independent replicates. (E) Growth curves of induced and non-induced epimastigotes expressing TcUBP1-GFP or GFP. At day 5 of culture, Tet was removed by washing and fresh medium was added. Values represent mean ± SD of 4 independent replicates. (F) Kinetoplast repositioning was evaluated by microscopy. Cells were stained with DAPI. (G) Staining of TS-SAPA by immunofluorescence in induced early-log epimastigotes expressing TcUBP1-GFP, and after Tet wash. DNA was stained with DAPI, shown in cyan. Images are representative of 3 independent experiments.

Metacyclogenesis is characterized by a massive reduction in translation rates that is concomitant with growth arrest, both *in vivo* and *in vitro* [44]. If TcUBP1 is a translational repressor that promotes metacyclogenesis, a growth arrest should be expected. Ectopic expression of TcUBP1-GFP showed a remarkable inhibition of parasite proliferation compared to non-induced cultures (Fig 6A). Growth curves derived from control GFP-expressing parasites showed that inducible expression of the transgene did not affect growth (Fig 6B). As an example of growth arrest induced by translation inhibition, we performed a growth curve of wt epimastigotes in the presence of CHX. The treatment with this translational inhibitor promoted growth arrest sooner than TcUBP1-GFP (Fig 6C). This could be explained by the expected delay of 24 h to reach the desired TcUBP1-GFP levels induced from the pTcINDEX vector.

To distinguish between parasite death and proliferation inhibition as a cause of TcUBP1-GFP reduced growth rates, we determined cell viability by incubating parasites with low concentrations of propidium iodide and analyzed them by flow cytometry at days 5 and 10 after Tet addition. We did not observe differences in the amount of dead cells in any of the time points between the studied cultures, indicating that TcUBP1-GFP did not induce cell death (Fig 6D). To test if global reduction of translation promotes differentiation from epimastigotes to metacyclic trypomastigotes, we incubated wt late logarithmic parasites with CHX for 5 days. We observed 86% of dead cells by propidium iodide incorporation (S8 Fig), suggesting that global translational inhibition leads to cell death and that it is not a driver for metacyclogenesis.

To analyze if TcUBP1-GFP-induced growth arrest was reversible, we removed Tet from induced cultures at day 5 by washing the cells, and evaluated parasite number, kinetoplast position and TS-SAPA staining for 10 more days (Fig 6E, 6F and 6G). In parasites where TcUBP1-GFP was induced, there was no recovery from the growth defect (Fig 6E). Control populations of induced GFP or uninduced TcUBP1-GFP showed no signs of growth defect. After 5 days from Tet addition, TcUBP1-GFP induced parasites showed 64% of cells with an orthogonal kinetoplast (Fig 6F), and internal staining with the anti-TS-SAPA antibody (Fig 6G). This staining is compatible with previous results using this antibody in epimastigotes undergoing metacyclogenesis [45]. After 5 days from Tet removal (day 10 of culture) the proportion of parasites with an orthogonal kinetoplast was 61% (Fig 6E). These parasites showed reduced TcUBP1-GFP fluorescence, while TS-SAPA staining was increased (Fig 6G). At 10 days from Tet removal (day 15 of culture), 53% of parasites still showed an orthogonal kinetoplast (Fig 6E), while there was no sign of TcUBP1-GFP fluorescence (Fig 6G). In contrast, staining with the anti-TS-SAPA antibody was still high. Overall, these results strongly suggest that the transient increase in TcUBP1 levels can promote the beginning of the metacyclogenesis process, which can not be reversed by returning TcUBP1 levels to the previous state, thus showing a commitment with differentiation.

### Low complexity regions of TcUBP1 are involved in development to infectivity

**Fig 7:**
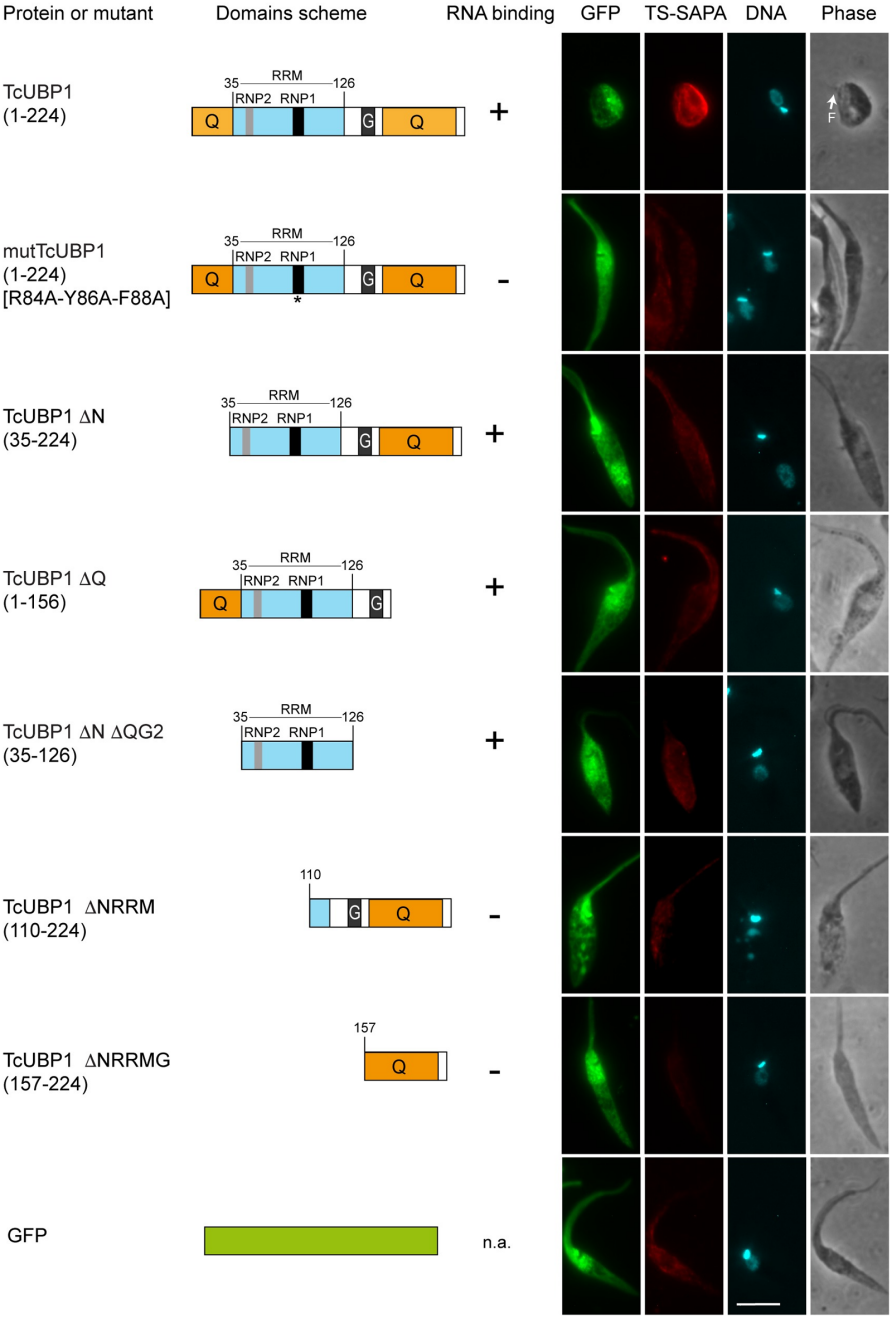
Cellular morphology and TS-SAPA expression in parasites ectopically expressing mutated or truncated forms of TcUBP1. Immunofluorescence of epimastigotes expressing the indicated TcUBP1-GFP constructs at day 10 of culture. *Trans*-sialidase expression was assessed using anti-TS-SAPA antibody and Alexa-568 goat anti-rabbit IgG. DNA was stained with DAPI shown in cyan. Images are representative of 3 independent experiments. Binding to RNA is based on previous evidence from our laboratory [19, 20]. Scale bars, 5 μm. Low complexity (LC) regions are shown in orange. RRM domain is shown in light blue.

TcUBP1 is a protein with modular architecture. A single central RRM provides mRNA binding and target specificity, flanked by N and C-terminal LC sequences, composed by 38 and 43% glutamines, respectively (Fig 7) [24]. N and C-terminal LC sequences seem to be intrinsically disordered structures, whose structure could not be determined earlier [46]. In order to determine the influence of the different TcUBP1 domains and regions on the parasite differentiation phenotype, we ectopically expressed different protein mutants and fragments fused to GFP (Fig 7). We analyzed TS-SAPA staining, the presence of an orthogonal kinetoplast and the drop-like phenotype in early logarithmic epimastigotes expressing these constructs. As expected, full length TcUBP1-GFP showed all the hallmarks previously analyzed. A triple point RNP1 RRM mutant (mutTcUBP1), which was previously shown to be unable to bind to RNA or to be recruited to mRNA granules [19], did not show any of the novel phenotypes of unmodified TcUBP1. This suggests that TcUBP1-induced differentiation is dependent on binding to RNA. Moreover, TcUBP1 fragments lacking the N (ΔN) or C-terminal (ΔQ) glutamine-rich LC sequences, did not resemble the effects exerted by TcUBP1 (Fig 7). These two constructs contain the functional RRM of TcUBP1, can bind to RNA *in vitro* [19, 47], and can be recruited to mRNA granules [17]. Thus, a functional RRM together with a single LC sequence, either on its N or C-terminal end, is not enough to promote full commitment to the differentiating phenotype. The smallest portion of TcUBP1 with the capability to bind to RNA (ΔNΔQG2), corresponding to the RRM, was also unable to reproduce the effects of full length TcUBP1. The complete C-terminal region, comprising the C-terminal LC sequence, or the C-terminal LC sequence alone, did not show any difference to control GFP-expressing parasites (Fig 7). These results show that only full length TcUBP1 is able to promote the differentiation phenotype. Therefore, removal of low complexity sequences, with previous unknown function, makes TcUBP1 dysfunctional to reproduce this novel phenotype. To sum up, we propose that TcUBP1 engages in multiple interactions involving N and C-terminal Q-rich LC domains while bound to RNA.

## Discussion

It is well established that post-transcriptional mechanisms have a huge impact on trypanosome regulation of gene expression [48]. These mechanisms also dictate the developmental transformation trypanosomes suffer during differentiation from non-infective to infective forms, as a response to environmental stimulus [49]. Current tools allow the analysis of mRNA and proteins that are up or downregulated as a response to external differentiation signals. This is the case of RBP6 in *T*. *brucei* [9], whose coding mRNA was found to be increased by a 13-fold in trypanosomes from the proventriculus. By overexpressing TbRBP6 in cultured noninfectious procyclic trypanosomes, the developmental stages that are observed in *tse tse* fly were recapitulated, including the generation of infective metacyclic forms expressing the variant surface glycoprotein [9]. However, most mRNAs or proteins that are upregulated in this manner typically correspond to surface proteins or virulence factors associated to the final developmental form [50, 51]. Also, In *T*. *brucei*, the CCCH Zinc Finger protein TbZFP1 is transiently elevated during differentiation from bloodstream to procyclic trypomastigotes forms, while its genetic ablation compromises the parasite differentiation program [10, 11]. It was also demonstrated that the ectopic expression of TbZFP2 results in a dramatic procyclic stage-specific remodeling of the trypanosome, similar to the morphogenic events of differentiation [11]. Additionally, forced TbRBP10-expression in procyclics induced a switch towards bloodstream-form mRNA expression patterns, with concomitant growth inhibition. Tethering of TbRBP10 to a reporter mRNA inhibited translation, and halved the abundance of the bound mRNA [12]. More recently, it has been shown that TbRBP10 expression promotes the differentiation to the bloodstream state [16]. These lines of evidence support the role of RBPs as potential master regulators of differentiation in trypanosomes. However, data for RBPs promoting differentiation is completely lacking *in T*. *cruzi*.

In this work, we show that TcUBP1 is enriched during metacyclogenesis in *T*. *cruzi*. Previously, De Godoy and coworkers also detected a rise in TcUBP1 levels during metacyclogenesis in a quantitative proteomic approach [28]. TcUBP1 associates to RNA secondary structures rather than to specific sequences [21], being these structures highly represented in a multitude of mRNAs, including transcripts coding for surface proteins, metabolism enzymes and protein kinases [21, 52]. This would imply that it can associate to many different transcripts, thus supporting a role for TcUBP1 as a putative regulator of post-transcriptional gene expression. Indeed, ectopic expression of TcUBP1-GFP in early-log epimastigotes cultures showed several hallmarks of parasites undergoing differentiation to metacyclic trypomastigotes. During developmental differentiation of epimastigotes to infective non-replicating metacyclic trypomastigotes the kinetoplast is repositioned to the posterior end of the parasite [25, 53]. The kinetoplast transits beside the nucleus, during which it can be found in an orthogonal position respective to the axis formed by the flagellum and nucleus in intermediate developmental forms (S4A Fig). Kollien and Schaub adopted the term drop-like morphology for forms found in the rectum of *Triatoma infestans* starved insects infected with *T*. *cruzi* [26]. Drop-like forms could be easily detected as soon as 48 hours after TcUBP1-GFP induction. It takes 24 hours for TcUBP1-GFP to be expressed, so we can conclude that the first emerging drop-like forms acquire this morphology 24 hours after TcUBP1 (endogenous plus ectopic) achieves a critical level to initiate translational repression. After the first 48 hours of TcUBP1-GFP induction drop-like forms accumulate, reaching more than half of the population by day 5. Drop-like parasites derived from wt *in vitro* differentiated parasites in TAU medium (S4C Fig) emerge as soon as 24 hours after incubation. If we compare these two results we conclude that TAU induced metacyclogenesis is a much stronger inducer of metacyclogenesis, although both stimulus seem to operate at similar rate once a critical level of TcUBP1 is reached.

In parasites showing the drop-like phenotype we were able to detect TS-SAPA, a protein involved in infection, which is exclusively expressed by infective trypomastigotes of *T*. *cruzi*, but not by amastigotes or epimastigotes [29]. We were also able to determine that these parasites do not express TSSA, a surface protein exclusively expressed in bloodstream trypomastigotes [34]. These observations are consistent with the development towards the metacyclic form by TcUBP1-expressing parasites. The expression of TS-SAPA, but not TSSA, clearly argues against unregulated gene expression havoc induced by ectopic expression of this RBP. As an alternative to the use of antibodies to detect stage-specific surface molecules we evaluated the resistance to lysis by complement in drop-like forms. During the differentiation of epimastigotes to metacyclic trypomastigotes, parasites also undergo a physiological change that confers them the capacity to evade complement-mediated lysis [54]. Thus, epimastigotes are not protected from the lytic effects of the innate immune response, while metacyclic trypomastigotes can circumvent it. The increased resistance to complement-mediated lysis by TcUBP1-GFP-induced drop-like forms suggests that complement regulatory proteins become expressed during this morphological metamorphosis [33], preparing them for the encounter with an immune system.

Ectopic expression of TcUBP1 in late logarithmic epimastigotes, which is a culture condition where differentiation is enhanced [36], promoted spontaneous differentiation under LIT medium culture, and enhanced metacyclogenesis under induced differentiating conditions (TAU medium). Metacyclic trypomastigotes promoted by ectopic expression of TcUBP1-GFP are functional infective forms as shown by infection of VERO cells in culture (Fig 3C and 3D). This result provides an observer-independent way to determine the development of infective metacyclic trypomastigotes induced by TcUBP1, as compared to control cultures. Furthermore, the intracellular development of TcUBP1-induced metacyclics into amastigotes implies that the progression of the life cycle is not compromised. Thus, it is important to highlight the full functionality of these parasites regarding infection, amastigogenesis and replication.

Previously, we have used TcUBP1-GFP ectopic expression in *T*. *cruzi* epimastigotes to localize this RBP in mRNA granules [17], and to show that TcUBP1 is a nucleocytoplasmic shuttling protein [19]. In fact, it seems that cytoplasmic TcUBP1 levels are strictly regulated, since overexpression at high Tet concentrations (0.5 μg/ml) promotes nuclear accumulation of this RBP (S1 Fig), probably as a mechanism to compensate for the presence of free TcUBP1 levels in the cytoplasm. In fact, parasites induced with the higher Tet concentrations did not show the drop-like phenotype, suggesting that TcUBP1-GFP can only induce differentiation in the cytoplasm, where translation occurs, and not in the nucleus. However, never before we were able to notice the kinetoplast relocalization and the drop-like phenotype described in this work. This can be explained because of the differences in the way TcUBP1-GFP has been expressed in our previous works. Before, we used a constitutively expressing vector such as pTEX [55], which requires long antibiotic selection times. This selection process could have been selecting parasites that compensate TcUBP1-induced growth deficiencies, thus diluting and loosing parasites with the drop-like phenotype and with an orthogonal kinetoplast. In fact, pTEX-TcUBP1-GFP transgenic cultures usually contain a low number of parasites expressing TcUBP1-GFP, suggesting that these cells struggled to survive. Here, we have used the Tet-inducible pTcINDEX vector, with which we were able to induce the expression of TcUBP1-GFP in almost the whole population of cells at demand, at similar levels as with the pTEX vector. We can conclude that inducible ectopic expression in trypanosomes is a suitable experimental approach to raise the levels of an RBP and determine its effects on differentiation, phenotypic transformations and expression of differentiation-markers. However, attention should be paid to the levels of ectopic expression; if we had used the higher Tet concentration without previously knowing anything about TcUBP1, we would have concluded that TcUBP1 is a predominantly nuclear protein that does not have any effect on metacyclogenesis, which is not.

Recently, translational modulation has been proposed as a major influence on the resulting protein products in trypanosomes [56]. Proteomic and ribosome profiling approaches have shown numerous differences in protein abundance between developmental stages both in *T*. *cruzi* and *T*. *brucei* [57, 58]. Yet, translational modulation has been more elusive to determine, probably because it is difficult to confirm that a given protein is responsible for repressing/enhancing translation. Here, we have developed our own *in vivo T*. *cruzi* system to examine the function of any protein when tethered to a reporter 3’ UTR. The virtue of this system relies on its ability to determine if a protein factor affects mRNA abundance and/or translation efficiency. This analysis is performed independently of sequence preference and without the influence of other potential factors binding to neighboring RNA structures. This is the first time that an RBP-tethering system is used in *T*. *cruzi*. In *T*. *brucei* it has been previously used to determine that, as expected, PABP1 can increase mRNA abundance and translation when tethered to a reporter CAT mRNA, while RBP10 can promote decreased mRNA abundance and translation [12]. Unlike the *T*. *brucei* system, which used Northern blot for quantitation of mRNA molecules [12, 16], we have implemented the use of Real-Time RT-PCR for the quantification of the reporter and Neomycin resistance mRNAs. In our tethering system, TcPABP1 behaved in the same way as in *T*. *brucei*, thus validating our experimental approach. A similar system has been used in *T*. *brucei* to show that SCD6 has a role in translational repression [15]. Erben and coworkers have used this kind of experimental approach to identify many potential mRNA regulators with no previously annotated function, or functions unrelated to mRNA metabolism [39]. In our *in vivo* RBP tethering system we found that TcZFP3 behaved like an mRNA stabilizer and a translational repressor. Unfortunately, contrastable information for the function of this protein is lacking in *T*. *cruzi*. In *T*. *brucei*, ZFP3 has been proposed to enhance the translation of procyclin EP1 mRNA directly [59]. More recently, ectopic expression of TbZFP3 showed to increase the abundance of two reporter mRNAs bearing the *Rbp23* or *SmB* 3’ UTR [59]. However, this increase in mRNA abundance did not correlate with an increase in CAT activity, suggesting that ZFP3 could also stabilize and repress translation of mRNA [17]. This hypothesis is in clear agreement with the results we obtained for TcZFP3 in our tethering system. We would also like to highlight the potential function of TcRBP4 as an mRNA stabilizer, promoting the translation of the reporter mRNA. This function is the opposite of the one of TcUBP1, showing an unbiased output for the system.

Although mRNA granules from trypanosomes, which contain TcUBP1, accomplish translational repression in response to a stressful environmental condition, TcUBP1 potential as a translational repressor was never examined before. Previous work from our group proposed an mRNA destabilizing function for TcUBP1 [20], which was specific for SMUG mRNA and not for other mRNAs. Our new evidence does not rebut this previous hypothesis. The destabilizing effect of TcUBP1 could not be confirmed statistically in our tethering system, probably because TcUBP1 needs another context of protein factors in a RNP complex, which is probably found in SMUG 3’ UTR. Using our *T*. *cruzi* tethering system we were able to determine that TcUBP1 can significantly repress translation. Central to the interpretation of our observations was the notorious effect of TcUBP1-GFP ectopic expression in protein translation by using the SUnSET method [40]. This experimental approach was used and validated for the first time in trypanosomes in this work. It allowed us to confirm that TcUBP1-GFP expressing parasites are experiencing a reduced translation rate as compared to uninduced or GFP induced controls (Fig 5B). Although we did not compare its sensitivity to classic [35S]-Met incorporation methods, it is clear that non-radioactive methods provide ease of use, especially when working with pathogens. The translational repression induced by the ectopic expression of TcUBP1-GFP was confirmed by qualitatively analyzing the polysomes profile, showing that translation is reduced although not inhibited. This metabolic condition is clearly different from that found in starved parasites, which lack polysomes, show granules of TcDhh1 (mRNA granules) and do not differentiate [60].

Another evidence arguing for a translational repression function in TcUBP1 is the enhancement in the formation of mRNA granules under suboptimal starvation stress (Fig 5C and 5D). SCD6 was previously proposed as a translational repressor [15], promoting the formation of mRNA granules in *T*. *brucei* [43]. Unlike TcUBP1, TbSCD6 seems to promote the recruitment of silenced mRNAs into granules without affecting ongoing translation [43]. Although TcUBP1 did not enhance the number of granules per cell, it did promote an increase in the size of mRNA granules (Fig 5E and 5F). We can hypothesize that ectopic expression of TcUBP1 could be increasing the speed at which mRNA granules are formed by removing certain mRNAs from the translation pool, leading to a faster response to starvation with increased granule size as compared to control parasites.

Previously, TcUBP1-interacting mRNA targets were found to be enriched in genes coding for proteins involved in metabolism. From these transcripts, an RNA motif termed UBP1m was identified in the 3´ UTR of these mRNAs [21]. Accordingly, in a posterior *in silico* analysis UBP1m was shown to be enriched in transcripts coding for surface proteins, metabolism enzymes and protein kinases [52]. This could be interpreted as TcUBP1 being involved in a regulatory cascade, repressing the translation of several factors required to maintain the epimastigote life form, which in turn triggers metacyclogenesis. As such, the expression of TS-SAPA could be an indirect consequence of TcUBP1 ectopic expression. Unfortunately, these putative key differentiation inhibitors are unknown in *T*. *cruzi*.

The association between translational repression and differentiation in *T*. *cruzi* has been demonstrated before for the kinase TcK2. TcK2 might be acting as a sensor of cytoplasmic heme, being activated in its absence, thus phosphorylating eIF2α [61]. Phosphorylation of eIF2α is a known trigger to arrest growth and translation initiation in many cell types, including *T*. *cruzi* [35]. In consequence, this signal seems to potentiate the differentiation of epimastigotes to metacyclic trypomastigotes as a result of translation inhibition. It was likewise observed for TcUBP1-GFP expressing parasites that a severe defect in growth affected these cells (Fig 6). This growth defect could be recapitulated by inhibiting translation with CHX. In spite of this, translational inhibition with CHX did not promote differentiation of epimastigotes, suggesting that a developmental program which selectively represses the translation of specific factors should be triggered, either by eIF2α phosphorylation, by a rise in TcUBP1 levels, or by other unexplored mechanisms. TcUBP1-induced growth arrest, kinetoplast relocalization and expression of TS-SAPA proved to be irreversible after washing out Tet (Fig 6E, 6F and 6G). We can suggest that these parasites are committed with a developmental program that cannot be turned off. Our results are in accordance with Domingo-Samanes *et al*. work, who established that the differentiation process in *T*. *brucei* is an irreversible and unidirectional bistable switch, involving irreversible commitment steps; and that differentiation is independent of the signal after commitment [62].

Full length TcUBP1 is required for the progression into infectivity, an affirmation that is supported by the mutational analysis we performed over its whole primary structure (Fig 7). This conclusion is based upon several results. On one hand, it is clear that the differentiation progression is dependent on the binding of TcUBP1 onto mRNA targets; the mutant of the RNP1 octapeptide of the RRM could not recapitulate the effect of unmodified TcUBP1. We have previously shown that this mutant TcUBP1 RRM is unable to bind to RNA *in vitro*, and is also unable to associate to mRNA granules [19]. Furthermore, complete deletion of the RRM also rendered an effect-less protein, probably due to the incapacity of TcUBP1 LC sequences to bind to RNA [19]. On the other hand, and to our surprise, elimination of either the N or C-terminal LC sequences of TcUBP1 also prevented the differentiation effect observed for the full-length protein (Fig 7). As such, we adopted the provisional hypothesis that both LC sequences of TcUBP1 might be involved in the repression of translation, ultimately triggering the process of differentiation. LC sequences have been proposed to orchestrate the dynamic assembly of RNP granules in yeasts and mammalian cells [3]. One of the most emblematic cases of LC-containing RBPs is Fused in Sarcoma (FUS). FUS has been a model protein for the demonstration of the liquid-droplet nature of RNA granules [63]. However, it is not completely clear how translational repression can be modulated by RBPs containing LC domains, as we suggest here for TcUBP1. Current evidence indicates that LC sequences promote homo and heterotypic interactions for the development of liquid droplets and amyloid-like structures [64]. Interactions of this kind by TcUBP1 could be promoting translational repression by recruiting different mRNA-protein complexes, thus forming submicroscopic foci that promote silencing of the bound RNA. It is tempting to speculate that LC sequences might be the driving force for the biogenesis of the initial *puncta* that give birth to RNA granules [65].

In summary, we provide mechanistic evidence of the role of the RBP TcUBP1 on *T*. *cruzi* differentiation through translation repression. Together, our results deepen the knowledge of gene expression regulation that occurs during differentiation from non-infective to infective parasite forms.

## Materials and methods

### Parasite cultures, differentiation and starvation

*Trypanosoma cruzi* epimastigotes, strain CL Brener, were cultured in liver infusion tryptose (LIT) medium with 0.001% bovine hemin and 10% heat-inactivated fetal calf serum (LIT complete medium) at 28° C. Experiments were performed with cultures at early logarithmic phase of growth (1–2 x 10^7^/ml) or at late logarithmic phase of growth (5–6 x 10^7^/ml). To determine parasite densities, we used a hemocytometer without fixation. Transgenic parasites containing the Luciferase reporter in pTEX vector were maintained in 200 μg/ml G418.

For the differentiation into metacyclic-trypomastigotes we essentially followed the protocol described by Bonaldo et al. [66]. Briefly, cultures of epimastigotes at 5 x 10^7^ cells/ml were collected by centrifugation and resuspended at a density of 5 x 10^8^ cells/ml in Triatomine Artificial Urine (TAU) medium [190 mM NaCl, 17 mM KCl, 2 mM MgCl_2_, 2 mM CaCl_2_, 8 mM phosphate buffer (pH 6.0)]. After 2 h of incubation at 28 °C the cells were diluted to 5 x 10^6^ per ml in TAU supplemented with 10 mM L-proline, 50 mM L-glutamic acid, 2 mM L-aspartic acid and 10 mM glucose and incubated at 28 °C for 96 h.

For tethering assays, transfected parasites were cultured in agitation at 28 °C at a density of 3 x 10^7^ parasites/ml.

For starvation studies, parasites were previously washed twice with PBS. Optimal starvation stimulus was defined as incubating late logarithmic epimastigotes cultures (5–6 x 10^7^/ml) in PBS (plus Tet for induced cultures) for 24 h. Suboptimal starvation stimulus was defined as incubating mid logarithmic epimastigotes cultures (3 x 10^7^/ml) in PBS (plus Tet for induced cultures) for 24 h. To analyze the percentage of parasites with mRNA granules a minimum of 200 cells were analyzed per culture through 5 different experiments. To define the size of granules, the area of the biggest granule from 20 different parasites for each culture was measured using ImageJ. To analyze the number of granules per cell, 40 parasites with mRNA granules were analyzed.

For time-course growth curves, a culture of 3 x 10^7^ per ml was used as a starter inoculum for the initial culture starting at 5 x 10^6^ parasites/ml [67]. Cultures were incubated in agitation at 28 °C in the presence or absence of Tet or cycloheximide.

For recovery studies, induced cultures were washed with fresh media 5 days before the addition of Tet, and allowed to resume growth for 5 more days in complete LIT medium without Tet.

### TcUBP1 ectopic expression

For the construction of the pTcINDEX-GFP vector we PCR-amplified the GFP open reading frame (ORF) using Primers GFP Fwd BamHI (cgggatccCATGGTGAGCAAGGGCGAGGAGC) and GFP Rev BglII (cgagatctTTACTTGTACAGCTCGTCCATGCC) from pTEX-GFP vector [17]. After digesting this insert with BamHI and BglII, it was cloned into the BamHI site of pTcINDEX [23]. After checking the orientation of different clones by PCR, a clone with the GFP ORF in the correct orientation was selected for sequencing. This clone contains the GFP ORF flanked by a BamHI site in its 5’ end, while the BamHI at the 3’ end is destroyed by ligation with the BglII end. After BamHI digestion and removal of 5’ phosphates in the resulting pTcINDEX vector, we subcloned the full length TcUBP1 ORF, or its RNP1 mutant and several fragments that were previously cloned into the BamHI site of the pTEX-GFP vector [17, 19].

Transfection and selection of parasites was essentially performed as previously described [23]. Briefly, epimastigotes were first transfected by electroporation with circular pLEW13 DNA, after which they were selected in complete medium containing 200 μg/ml G418. Stably transformed parasites, obtained after six weeks, were re-transfected with pTcINDEX-GFP constructs and selected with 100 μg/ml Hygromycin, together with G418. Induction of recombinant proteins from the pTcINDEX-GFP vector was performed by the addition of Tet at 0.05 μg/ml final concentration, or otherwise stated. Parasites reached the desired induction levels after 24 h of Tet addition. For incubation times longer than five days, Tet was re-added at 0.05 μg/ml at day five of the time curve.

### Immunoblotting

For all protein extracts, parasites were first washed once with PBS. For Western blot, parasites were lysed for 15 min in TBS plus 0.5% NP-40, with the addition of 100 μM trans-Epoxysuccinyl-L-leucylamido (guanidino) butane (E64) and 1 mM Phenylmethylsulfonyl Fluoride (PMSF) (Lysis Buffer). After lysis, samples were centrifuged at 10,000 g for 10 min at 4° C. Sample Buffer (Final concentration: 50 mM Tris-HCl pH 6.8; 2% Sodium Dodecyl Sulfate, SDS; 10% Glycerol; 0.02% Bromophenol Blue; 100 mM Dithiothreitol), was added to the supernatant and immediately boiled for 3 min. Afterwards, samples were treated with DNase I (Sigma) for 15 min at room temperature to reduce DNA viscosity. For Western blot, samples were loaded onto 10% SDS-polyacrylamide gels. Gels were transferred to Immobilon-NC transfer membranes (Millipore), probed with rabbit anti-TcUBP1 [21], mouse anti-GFP (Roche), mouse anti-Flag (Sigma), rabbit anti-puromycin (a kind gift from Dr. Walter from UCSF), and mouse anti-tubulin (Sigma). Rabbit anti-TS-SAPA recognizes a 12 amino acid repeat denominated Shed Acute Phase Antigen (SAPA), which is in the C-terminal part of the molecule, was a kind gift from Dr. Campetella from UNSAM [68]. We used IRDye secondary anti-rabbit antibody and anti-mouse antibodies (LI-COR). Detection was performed using the Odyssey Imaging System (LI-COR). Quantitation of protein bands was performed using Image Studio Lite software (LI-COR).

### Flow cytometry

Parasites from uninduced or Tet induced cultures (48 hs) were washed with PBS. Cells (1 x 10^5^) were analyzed in a CyFlow space cytometer (Partec, Germany).

For determination of the percentage of non-viable cells in parasite cultures, cells were washed in cold PBS and then resuspended in PBS supplemented with 2 mM glucose. After that, 1 μg/ml propidium iodide (PI) was added 5 minutes before FACS analysis as previously described [69]. The percentage of PI stained cells was analyzed in a CyFlow space cytometer (Partec, Germany).

### Immunostaining, mRNA fluorescence in situ hybridization and microscopy

Parasites were washed with PBS and attached to glass slides pretreated with 0.01% Poly-L-lysine for 30 min. The excess of parasites was removed and the slides were incubated with 4% paraformaldehyde in PBS for 20 min, washed with PBS and permeabilized with 0.2% saponin. Fixed and permeabilized cells were washed and incubated with primary antibody diluted in PBS supplemented with 2% BSA for 1h at room temperature. The slides were washed with PBS, incubated with Alexa 568 goat anti-rabbit IgG (Life technologies), washed with PBS and mounted in the presence of 10 μg/ml of 4-6-diamidino-2-phenylindole (DAPI). Fluorescence In Situ Hybridization (FISH) of mRNA was performed essentially as described [17]. Microscopic analyses were performed in an Eclipse E600 Microscope (Nikon, Tokyo, Japan) coupled to a SPOT RT color camera (Diagnostic Instruments). To determine the percentage of parasites with kinetoplast repositioning, we analyzed 239 cells for TcUBP1-GFP and 103 for GFP. For TS-SAPA expression determination, we used anti-TS-SAPA. To determine the percentage of spontaneous metacyclic trypomastigotes we analyzed 250 cells for TcUBP1-GFP and for GFP each time in four independent experiments.

### Complement-mediated lysis

Transfected epimastigotes were grown to a concentration of 2 x 10^7^/ml, induced or not with Tet for five days, washed once in PBS, and resuspended at 4 x 10^6^/ml in PBS. To analyze complement-mediated lysis, 10% human serum was added. Cells were incubated at 28°C and the number of motile parasites was determined by cell count at 10, 20, 30 and 60 min.

### SUnSET method

To monitor protein synthesis, we used the SUnSET method [40]. Briefly, puromycin is an aminonucleoside antibiotic produced by *Streptomyces alboniger*. It is a structural analog of aminoacyl tRNAs, which is incorporated into the nascent polypeptide chain and prevents elongation. When used in minimal amounts, puromycin incorporation in neosynthesized proteins reflects directly the rate of mRNA translation *in vitro*.

Wt parasites were incubated with CHX (50 μg/ml) or vehicle for 30 minutes at 28° C and then with puromycin (10 μg/ml) or vehicle for 30 minutes at 28° C. After that, parasites were washed and lysed. Puromycin incorporation was determined by Western blot using an anti-puromycin antibody. Protein loading was monitored using an anti-tubulin antibody. Transfected parasites were cultured for 5 and 10 days. After that, 3 x 10^7^ parasites were incubated with puromycin (10 μg/ml) or vehicle for 30 minutes at 28° C. Parasites were washed and lysed. Puromycin incorporation was determined by Western blot using an anti-puromycin antibody. Protein loading was monitored using an anti-tubulin antibody. Western blots were performed using secondary antibodies coupled to Alexa 800 and Alexa 680. To determine fluorescence intensity blots were analyzed using LI-COR software.

### Polysome analysis in sucrose gradients

For each gradient we used 5x10^8^ parasites. When indicated, parasites were induced with Tet (0.05 μg/ml) for 5 days. Before harvesting, cells were incubated with CHX (100 μg/ml) to prevent ribosomal run-off, or EDTA (10 mM) to dissociate ribosomal subunits, for 30 min., after which were washed in PBS containing CHX or EDTA, respectively. Parasites were lysed at 2,5x10^6^ cells/μl in Polysome buffer (20 mM Tris-HCl pH 7.6, 120 mM KCl, 5 mM MgCl_2_) supplemented with 0.5% NP-40, 1mM PMSF, 100 μM E64, 80U/ml RNasin (Promega), and 100 μg/ml CHX or 10 mM EDTA. After clearing by centrifugation at 20,000 g (10 min, 4° C), the soluble lysate was layered on a linear 15–50% sucrose gradient (12 ml), prepared in polysome buffer. Gradients were centrifuged for 2,5 hours at 35.000 rpm in a Beckman SW41Ti rotor at 4° C. Absorbance at 254 nm was monitored by using the UV detector of a ÄKTA HPLC system and pumping the gradient from bottom to top using a peristaltic pump.

### *In vitro* infection assays

For infection assays we used late logarithmic parasites expressing GFP or TcUBP1-GFP, which were induced with Tet for 5 days. After 5 days, there was a mixture of epimastigotes and metacyclic trypomastigotes in the TcUBP1-GFP culture. Cell invasion assays were carried out by seeding these cultures of parasites (2 x 10^7^ total cells) onto each well of 24-well plates containing 13-mm diameter round glass coverslips coated with 1 x 10^4^ VERO (African green monkey kidney) cells (American Type Culture Collection, VA). The medium was removed every 24 h for 72 h. After that, wells were washed with PBS and incubated with 4% paraformaldehyde in PBS for 20 min. Following extensive washing in PBS, cells were incubated for 10 min with NH_4_Cl and blocked for 1 h in PBS containing 2% (w/v) BSA and 2% (v/v) normal goat serum. Extracellular (attached) parasites were labeled with the addition of a *T*. *cruzi*-infected mouse serum followed by Alexa Fluor 568 conjugated secondary antibody (Invitrogen). Intracellular parasites were subsequently labeled with the addition of a *T*. *cruzi*-infected rabbit serum (diluted in PBS with 0.5% saponin) followed by Alexa Fluor 488 conjugated secondary antibody (Invitrogen). The coverslips were washed and mounted in the presence of 10 μg/ml of 4-6-diamidino-2-phenylindole (DAPI). Microscopic analyses were performed in an Eclipse E600 Microscope (Nikon, Tokyo, Japan) coupled to a SPOT RT color camera (Diagnostic Instruments).

### Tethering assays—DNA construction

For this construction, we used pTEX vector [55] and the Lambda (λ) bacteriophage antiterminator protein N (λN) peptide strategy to tether proteins to RNAs [37]. The RNA binding domain of the λN is used to tag the RBP. The reporter mRNA includes the sequence that codes for Firefly luciferase, which was obtained from the pGL3-Basic vector (Promega). Specific sequences of 19 nt (GGGCCCUGAAGAAGGGCCCUUUCCUUU) that are binding sites for λN (boxB) were inserted into the target RNA. These were artificially synthesized (Macrogen). To generate λN fusion proteins a synthetic sequence that codes for λN domain was designed (MDAQTRRRERRAEKQAWKAANGGS) (Macrogen). The last three amino acids (GGS) serve as a flexible union between λN domain and the fusion partner. Also, a FLAG tag, a small multiple cloning site and 3 stop codons in each frame were added following the λN coding-sequence. This allowed us to assess the expression of the fusion protein by Western blot, and clone the RBPs as λN-fusion proteins. The intergenic region between the *T*. *cruzi* actin I and II genes was used between the boxB sites and the λN-coding cistron. This sequence provides a stable 3´UTR for the reporter mRNA and sites for post-transcriptional processing for the reporter mRNA and for the sequence that codes for the λN-fusion protein. In this manner, using pTEX vector we could express the reporter mRNA that codes for luciferase reporter mRNA and also the λN-fusion proteins (Fig 4A). The major advantage of this system derives from the small size of the λN peptide and its target sequence, preventing interferences with the fused protein.

Cloning of GFP, TcPABP and TcUBP1 into pTEX-luciferase vector was performed by subcloning from the pTEX-GFP vector [67] using the enzyme BamHI for TcPABP and TcUBP1 and the enzymes BamHI and HindIII for GFP. For the cloning of TcRBP4 we PCR-amplified the open reading frames (ORF) using Primers Fwd BamHI (ggatccATGCGGAGTCGGAGCAGC) and Rev BamHI (ggatccCGTTCCTTCTCTTCTTCGTCCA). For TcZFP2 we PCR-amplified the open reading frames (ORF) using Primers Fwd BamHI (ggatccATGTCCTACCCGAATCGTTATG) and Rev HindIII (aagcttTCACTGGGTCTGTGCGGGC). For TcZFP3 we PCR-amplified the open reading frames (ORF) using Primers Fwd BamHI (ggatccATGCAGGGGTATTTTGCACTC) and Rev HindIII (aagcttTTATGACGCCGGCGTTTCTC). After checking the orientation of different clones by PCR, clones with the mentioned ORFs in the correct orientation were selected for sequencing.

### Luciferase assays

Luciferase activity was determined using the kit Bright-Glo Luciferase Assay System (Promega). Briefly, 1.5 x 10^7^ parasites were lysed and luciferase activity was measured following the kit instructions. Luminescence was normalized to the amount of protein present in the sample determined by the Bradford assay.

### RNA isolation and RT-qPCR

Total RNA was isolated from uninduced and induced parasites with Trizol Reagent according to manufacturer’s instructions (Life Technologies). Total RNA was purified using Direct-zol RNA MiniPrep columns (Zymo Research). RNA integrity was evaluated by 1% agarose gel electrophoresis. Samples were incubated with RQ1 DNAse (Promega) followed by enzyme inactivation. First strand cDNA was synthesized from total RNA samples using Superscript II reverse transcriptase (Life Technologies), following manufacturer´s instructions.

Real time quantitative PCR (qPCR) was performed using Kapa Sybr Fast Universal Kit (Biosystems) with primers described below. Luciferase mRNA abundance was measured with the oligonucleotides F 5’-CAATGGGACGTATGGGACAT-3’ and R 5’-TCGTCTGTCACCACCCATAG-3’. Neomycin resistance gene (Neo) mRNA was measured with oligonucleotides F 5’-TTTTTGACCGTGGTGGGAGG-3’ and R 5’-TTGATCCCTTCTCACAGCGG-3’. The reactions were carried out with the 7500 Real Time PCR System from Applied Biosystems.

### Data analysis for tethering assays

After selecting stably expressing parasite populations, we used real time RT-PCR to determine luciferase reporter and Neo mRNA levels, while protein levels were determined by Western blot using anti-Flag and anti-tubulin antibodies. Luciferase activity was determined by luminescence and total protein was measured by Bradford assay.

Luciferase activity was normalized to the amount of protein in the sample determined by Bradford (L), RBP protein levels was normalized to tubulin protein levels (P) and luciferase mRNA levels was normalized to the Neomycin resistance gen mRNA (Neo) levels contained in the pTEX vector (RNA). The abundance (A) of the reporter mRNA was established as the quotient between RNA and P. The translation efficiency (T) of the reporter mRNA was established as the quotient between luciferase activities (L) normalized to RNA abundance (RNA) and the amount of RBP (P) (Fig 4B).

### Statistical analyses

Data analysis was performed with GraphPad Prism 6.0 (GraphPad Software, La Jolla, CA, USA). Statistical differences were assessed by analysis of variance (ANOVA) with Tukey post-hoc analysis for multiple comparisons or with Dunnett post-hoc analysis for multiple comparisons to one control group. Differences with a p-value <0.05 were considered significant. For tethering assays data analysis, folds increase over 2 vs. control was considered significant.

## Supporting information

S1 Fig TcUBP1-GFP cellular localization after induction with different concentrations of Tet. Parasites were induced with the indicated concentration of Tet for 96 hs. Localization of TcUBP1-GFP is shown in transfected parasites. DNA was stained with DAPI. Scale bar, 5 μm.
(TIF)

S2 Fig Kinetoplast repositioning in TcUBP1-GFP expressing parasites. Localization of TcUBP1-GFP and GFP is shown in transfected parasites. DNA was stained with DAPI. Scale bar, 5 μm.
(TIF)

S3 Fig Individual measurements of FNK angles in induced epimastigotes expressing TcUBP1-GFP or GFP. (TIF)

S4 Fig *T*. *cruzi* morphology changes during differentiation from epimastigote to metacyclic trypomastigotes *in vitro*. (A). Wt parasites were incubated in TAU-3AAG culture medium for the indicated time periods. Details of the morphology of the most differentiated parasites at each time point, together with the movement of the kinetoplast to the posterior end of the cell. DNA was stained with DAPI. Scale bars, 5 μm. (B) Same as in A, showing fields with multiple parasites. DNA was stained with DAPI, and is shown in cyan merged with phase contrast images. Regular epimastigotes (highlighted orange) correspond to parasites predominant at 0 hours, drop-like epimastigotes (highlighted light blue) correspond to parasites predominant at 24 and 48 hours, metacyclic-like intermediate forms (highlighted grey) correspond to parasites that appear at 72 hours, and metacyclic trypomastigotes (highlighted gray) correspond to full differentiated parasites that appear at 96 hours. (C) Quantitation of phenotypes from B.
(TIF)

S5 Fig Induced ectopic expression of TcRBP35-GFP. Localization of TcRBP35-GFP (TcCLB.510661.230) is shown in transfected epimastigotes induced for 5 days. DNA was stained with DAPI, shown in cyan. A magnification of the indicated cell is shown. Scale bar, 5 μm.
(TIF)

S6 Fig TSSA expression in epimastigotes and trypomastigotes. (A) Immunofluorescent detection of TSSA in cell derived trypomastigotes. (B) Immunofluorescent detection of TSSA in GFP and TcUBP1-GFP induced epimastigotes. DNA was stained with DAPI. Scale bars, 5 μm.
(TIF)

S7 Fig *In vitro* infection with induced GFP expressing parasites. Seven days post-infection VERO cells were stained with mouse anti-*T*. *cruzi* before permeabilization (red) and with rabbit anti-*T*. *cruzi* after permeabilization (green). DNA was stained with DAPI, shown in cyan. Image is representative of 3 independent experiments.
(TIF)

S8 Fig Translational inhibition by cycloheximide leads to parasite death. Parasites were tested for incorporation of propidium iodide (PI) and analyzed by flow cytometry. Wt parasites were incubated with CHX at 50 μg/ml for five days. TcUBP1-GFP expressing parasites were analyzed five days after Tet addition. The dashed mark separates the populations considered to incorporate PI at the right.
(TIF)

S1 Table Individual measurements of FNK angles in induced epimastigotes expressing TcUBP1-GFP or GFP. (PDF)
