# Supplementary figures and images for: Translational repression by an RNA-binding protein promotes differentiation to infective forms in Trypanosoma cruzi
Source: PLoS Pathog. 2018 Jun 4;14(6):e1007059. doi: 10.1371/journal.ppat.1007059 (PMC6002132; doi:10.1371/journal.ppat.1007059)

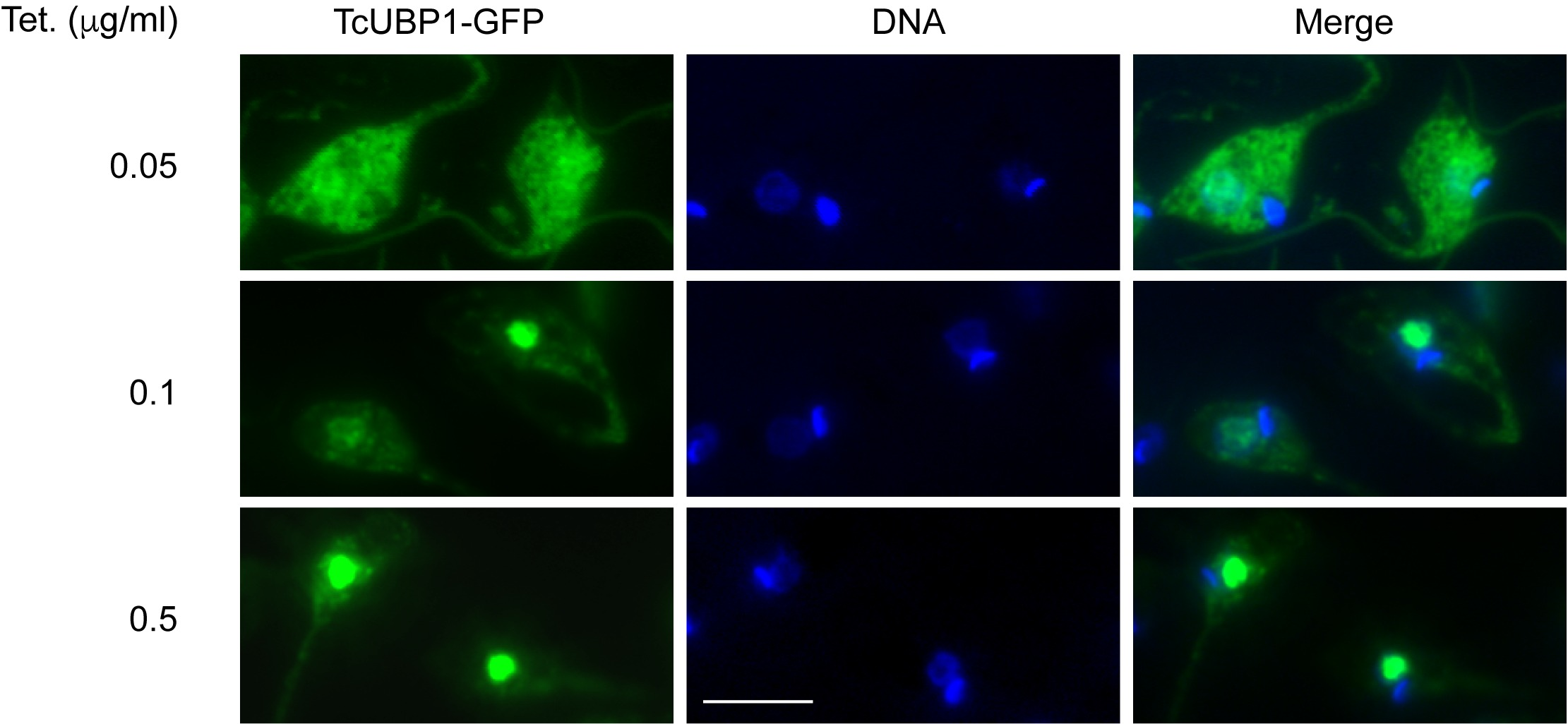

Supplement: S1 Fig — Parasites were induced with the indicated concentration of Tet for 96 hs. Localization of TcUBP1-GFP is shown in transfected parasites. DNA was stained with DAPI. Scale bar, 5 μm. (TIF) [file ppat.1007059.s001.tif]

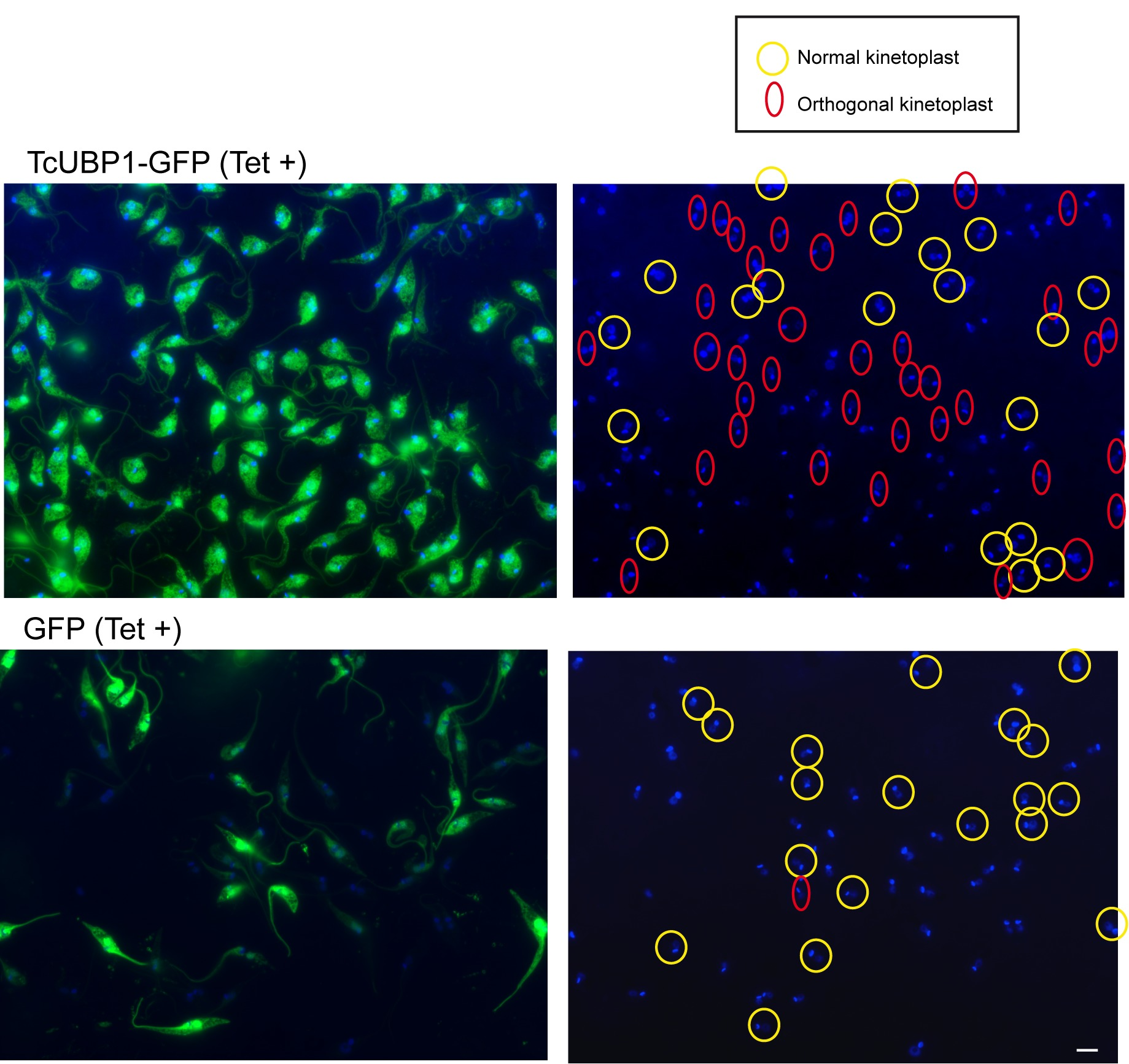

Supplement: S2 Fig — Localization of TcUBP1-GFP and GFP is shown in transfected parasites. DNA was stained with DAPI. Scale bar, 5 μm. (TIF) [file ppat.1007059.s002.tif]

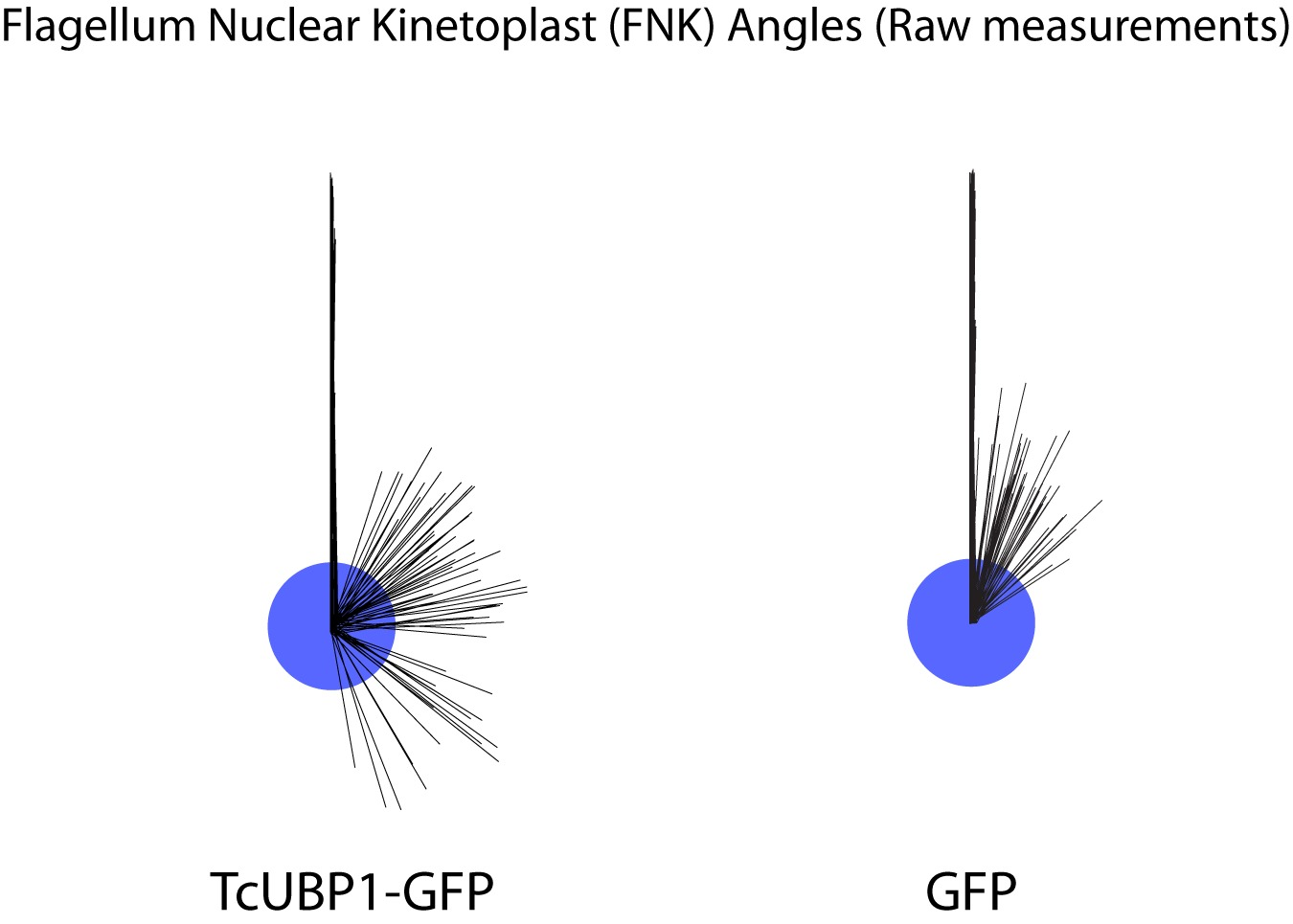

Supplement: S3 Fig — (TIF) [file ppat.1007059.s003.tif]

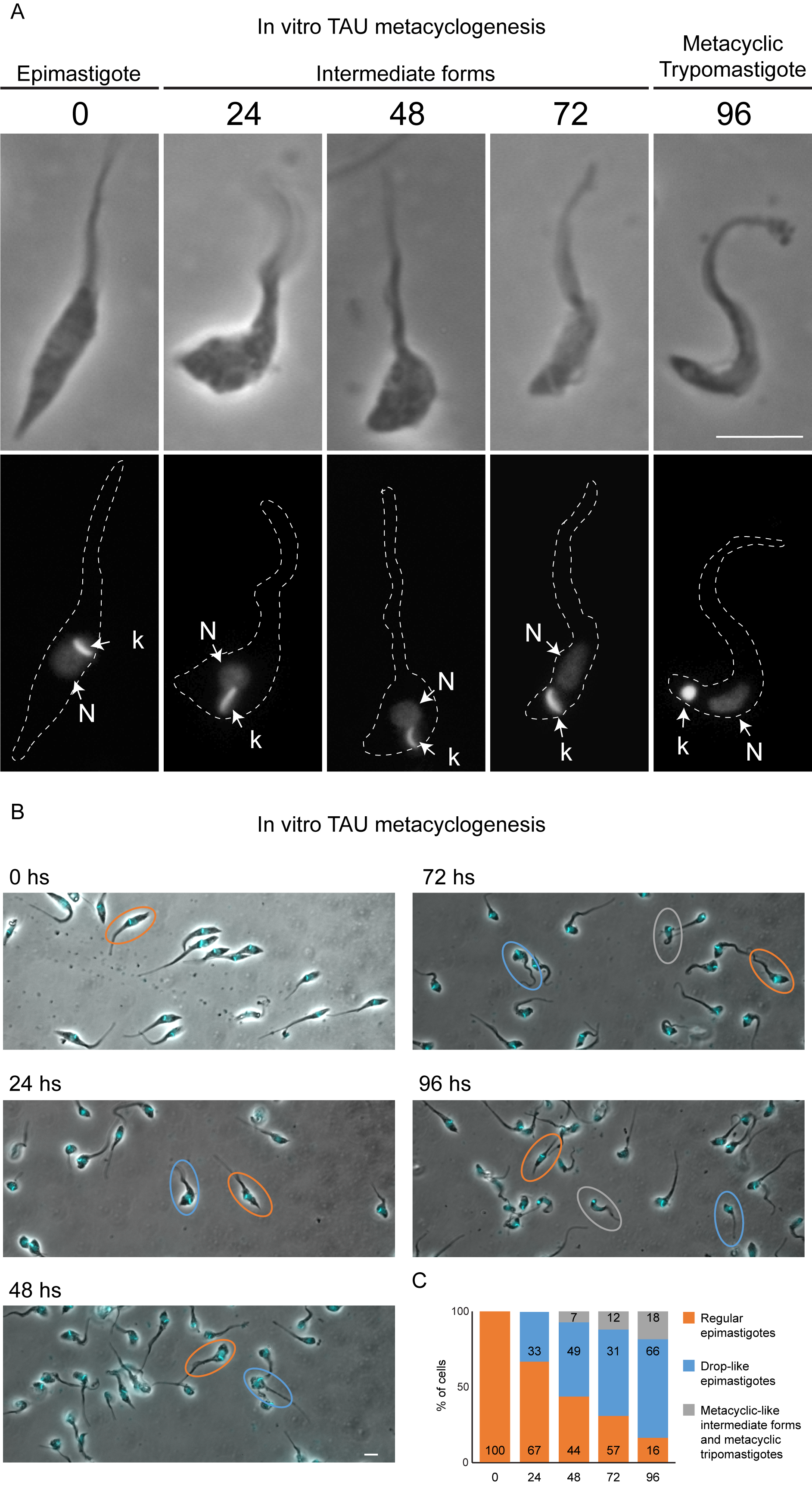

Supplement: S4 Fig — (A). Wt parasites were incubated in TAU-3AAG culture medium for the indicated time periods. Details of the morphology of the most differentiated parasites at each time point, together with the movement of the kinetoplast to the posterior end of the cell. DNA was stained with DAPI. Scale bars, 5 μm. (B) Same as in A, showing fields with multiple parasites. DNA was stained with DAPI, and is shown in cyan merged with phase contrast images. Regular epimastigotes (highlighted orange) correspond to parasites predominant at 0 hours, drop-like epimastigotes (highlighted light blue) correspond to parasites predominant at 24 and 48 hours, metacyclic-like intermediate forms (highlighted grey) correspond to parasites that appear at 72 hours, and metacyclic trypomastigotes (highlighted gray) correspond to full differentiated parasites that appear at 96 hours. (C) Quantitation of phenotypes from B. (TIF) [file ppat.1007059.s004.tif]

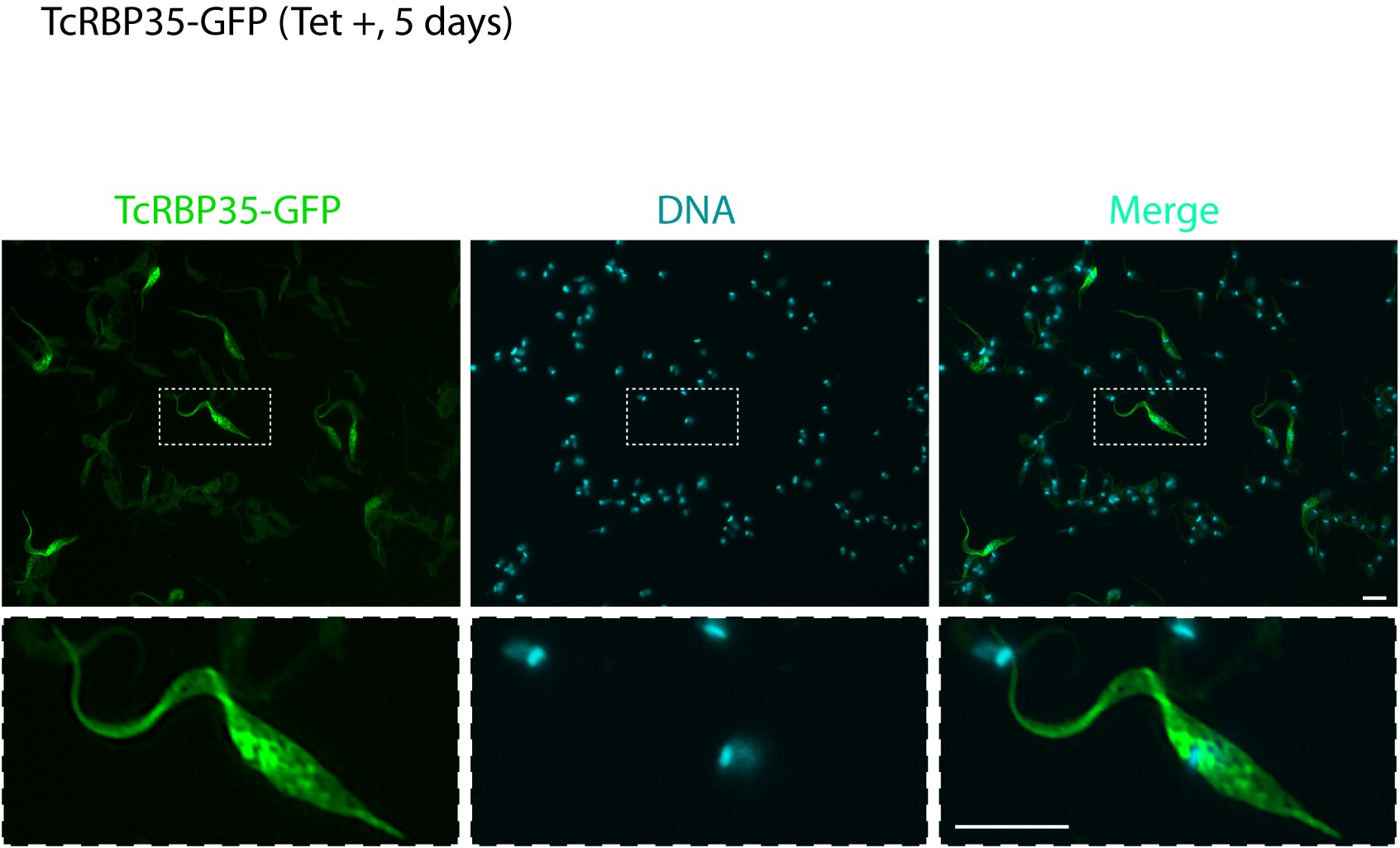

Supplement: S5 Fig — Localization of TcRBP35-GFP (TcCLB.510661.230) is shown in transfected epimastigotes induced for 5 days. DNA was stained with DAPI, shown in cyan. A magnification of the indicated cell is shown. Scale bar, 5 μm. (TIF) [file ppat.1007059.s005.tif]

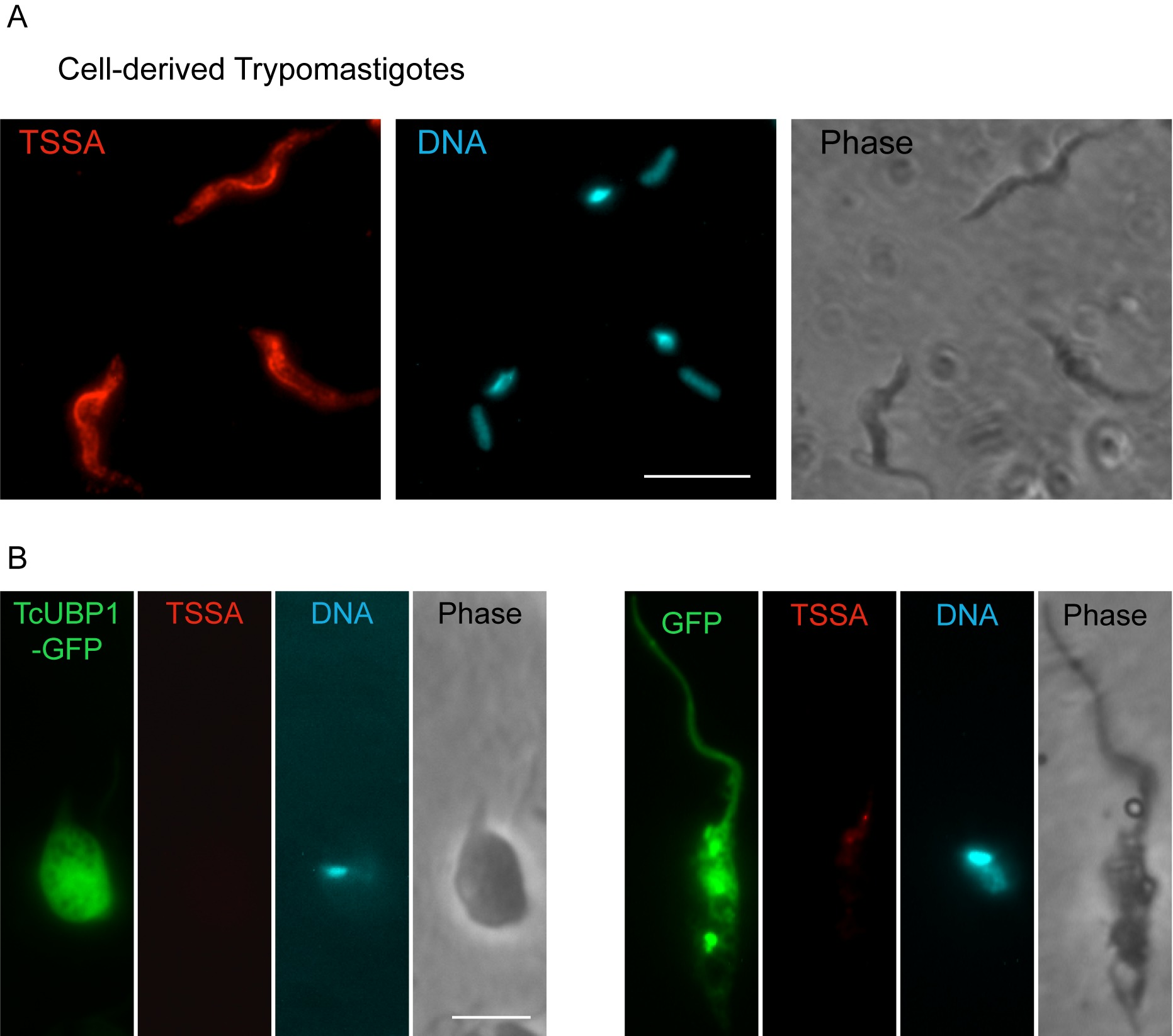

Supplement: S6 Fig — (A) Immunofluorescent detection of TSSA in cell derived trypomastigotes. (B) Immunofluorescent detection of TSSA in GFP and TcUBP1-GFP induced epimastigotes. DNA was stained with DAPI. Scale bars, 5 μm. (TIF) [file ppat.1007059.s006.tif]

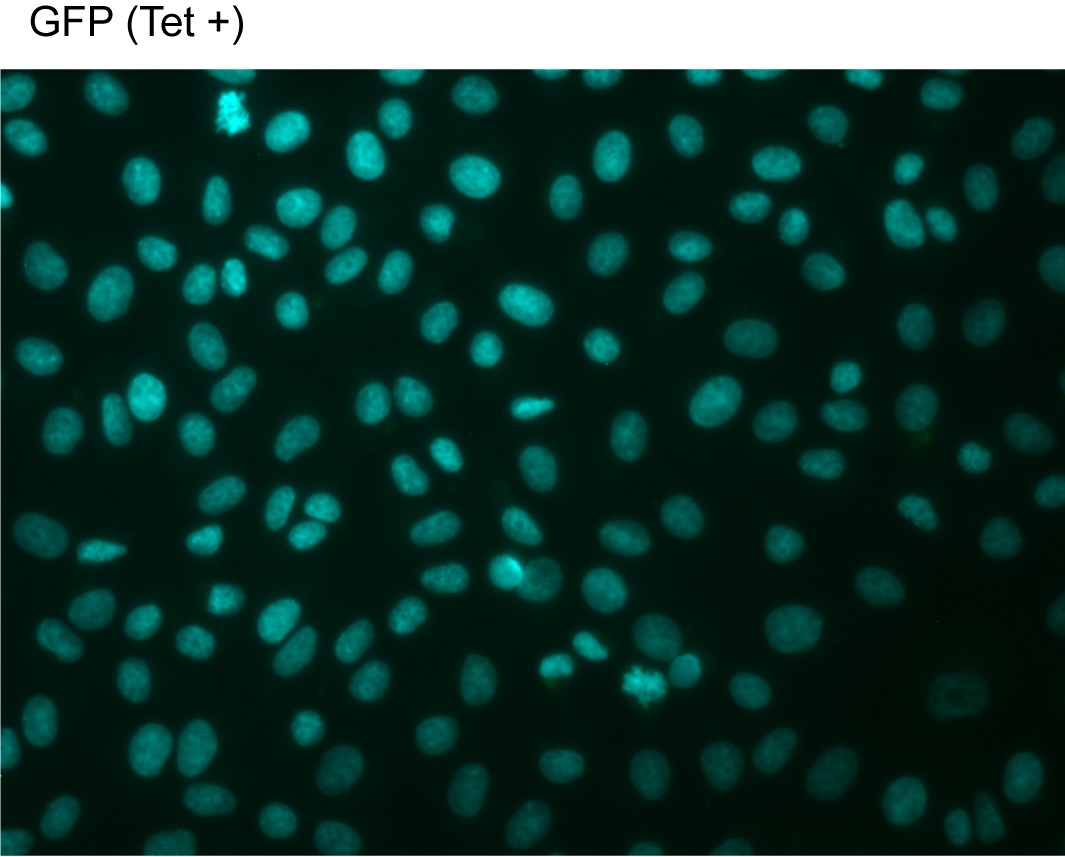

Supplement: S7 Fig — Seven days post-infection VERO cells were stained with mouse anti-T. cruzi before permeabilization (red) and with rabbit anti-T. cruzi after permeabilization (green). DNA was stained with DAPI, shown in cyan. Image is representative of 3 independent experiments. (TIF) [file ppat.1007059.s007.tif]

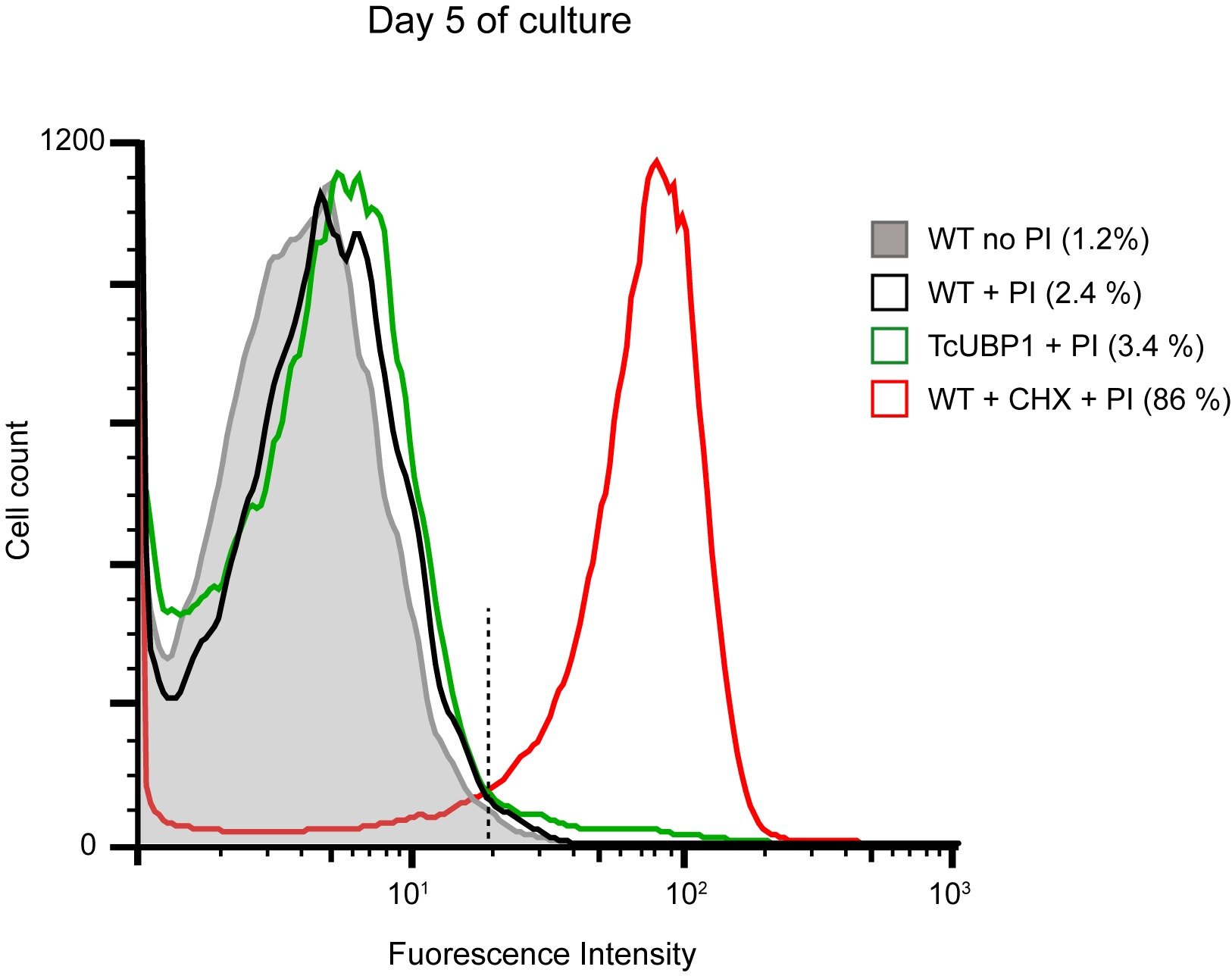

Supplement: S8 Fig — Parasites were tested for incorporation of propidium iodide (PI) and analyzed by flow cytometry. Wt parasites were incubated with CHX at 50 μg/ml for five days. TcUBP1-GFP expressing parasites were analyzed five days after Tet addition. The dashed mark separates the populations considered to incorporate PI at the right. (TIF) [file ppat.1007059.s008.tif]
