# Supplementary material for: Translational repression by an RNA-binding protein promotes differentiation to infective forms in Trypanosoma cruzi
Source: PLoS Pathog. 2018 Jun 4;14(6):e1007059. doi: 10.1371/journal.ppat.1007059 (PMC6002132; doi:10.1371/journal.ppat.1007059)
Supplement: S1 Table — (PDF) [file ppat.1007059.s009.pdf]

S1 Table. Individual measurements of FNK angles in induced epimastigotes expressing TcUBP1-GFP or GFP.

	TcUBP1-GFP	GFP
Parasite	FNK Angle	FNK Angle
1	76.26	55.89
2	81.28	16.69
3	87.21	30.61
4	30.75	32.77
5	49.42	43.20
6	81.15	24.29
7	110.09	21.26
8	64.31	15.95
9	44.24	20.39
10	67.28	16.17
11	167.32	10.29
12	88.28	16.83
13	112.03	49.55
14	75.75	15.31
15	34.35	22.05
16	77.91	21.52
17	25.87	17.43
18	55.99	40.44
19	123.91	7.40
20	49.33	2.74
21	147.80	35.96
22	158.04	14.19
23	122.38	1.62
24	141.05	10.69
25	46.57	28.23
26	23.41	47.81
27	51.20	7.87
28	62.12	18.35
29	92.71	28.54
30	125.09	0.56
31	97.26	47.78
32	121.96	19.36

	TcUBP1-GFP	GFP
Parasite	FNK Angle	FNK Angle
33	44.07	2.49
34	70.85	20.21
35	57.73	18.43
36	76.19	24.03
37	117.90	43.04
38	122.40	22.17
39	124.99	22.80
40	30.29	7.35
41	37.24	6.74
42	50.18	27.27
43	64.01	0.89
44	41.55	9.33
45	48.41	14.55
46	78.36	16.31
47	66.62	21.03
48	60.13	14.99
49	155.29	11.74
50	86.01	82.25
51	115.50	43.88
52	28.72	29.65
53	19.28	32.21
54	59.04	30.63
55	82.24	16.68
56	163.83	19.86
57	125.75	31.74
58	38.26	4.84
59	148.13	
60	72.08	
61	88.81	
62	44.01	
63	39.38	
64	148.46	
